# Mapping global evidence on strategies and interventions in neurotrauma and road traffic collisions prevention: a scoping review

**DOI:** 10.1186/s13643-020-01348-z

**Published:** 2020-05-20

**Authors:** Santhani M Selveindran, Tamara Tango, Muhammad Mukhtar Khan, Daniel Martin Simadibrata, Peter J. A. Hutchinson, Carol Brayne, Christine Hill, Franco Servadei, Angelos G. Kolias, Andres M. Rubiano, Alexis J. Joannides, Hamisi K. Shabani

**Affiliations:** 1 Department of Clinical Neurosciences, Addenbrooke’s Hospital, Cambridge, UK; 2 NIHR Global Health Research Group on Neurotrauma, University of Cambridge, Cambridge, UK; 3 Faculty of Medicine, University of Indonesia, Depok, Jawa Barat Indonesia; 4 Department of Neurosurgery, Northwest School of Medicine and Northwest General Hospital and Research Centre, Peshawar, Pakistan; 5 Institute of Public Health, University of Cambridge, Cambridge, UK; 6 Department of Neurosurgery, Humanitas University and Research Hospital, Milan, Italy; 7 World Federation of Neurosurgical Societies, Nyon, Switzerland; 8 Department of Neurosurgery, Universidad El Bosque, Bogota, Colombia; 9 Neurological Surgery Unit, Muhimbili Orthopaedic Institute and Muhimbili University College of Allied Health Sciences, Dar es Salaam, Tanzania

**Keywords:** Neurotrauma prevention, Road traffic collisions prevention, Preventative strategies and interventions, Low- and middle-income countries, High-income countries, Contextual factors

## Abstract

**Background:**

Neurotrauma is an important global health problem. The largest cause of neurotrauma worldwide is road traffic collisions (RTCs), particularly in low- and middle-income countries (LMICs). Neurotrauma and RTCs are preventable, and many preventative interventions have been implemented over the last decades, especially in high-income countries (HICs). However, it is uncertain if these strategies are applicable globally due to variations in environment, resources, population, culture and infrastructure. Given this issue, this scoping review aims to identify, quantify and describe the evidence on approaches in neurotrauma and RTCs prevention, and ascertain contextual factors that influence their implementation in LMICs and HICs.

**Methods:**

A systematic search was conducted using five electronic databases (MEDLINE, EMBASE, CINAHL, Global Health on EBSCO host, Cochrane Database of Systematic Reviews), grey literature databases, government and non-government websites, as well as bibliographic and citation searching of selected articles. The extracted data were presented using figures, tables, and accompanying narrative summaries. The results of this review were reported using the PRISMA Extension for Scoping Reviews (PRISMA-ScR).

**Results:**

A total of 411 publications met the inclusion criteria, including 349 primary studies and 62 reviews. More than 80% of the primary studies were from HICs and described all levels of neurotrauma prevention. Only 65 papers came from LMICs, which mostly described primary prevention, focussing on road safety. For the reviews, 41 papers (66.1%) reviewed primary, 18 tertiary (29.1%), and three secondary preventative approaches. Most of the primary papers in the reviews came from HICs (67.7%) with 5 reviews on only LMIC papers. Fifteen reviews (24.1%) included papers from both HICs and LMICs. Intervention settings ranged from nationwide to community-based but were not reported in 44 papers (10.8%), most of which were reviews. Contextual factors were described in 62 papers and varied depending on the interventions.

**Conclusions:**

There is a large quantity of global evidence on strategies and interventions for neurotrauma and RTCs prevention. However, fewer papers were from LMICs, especially on secondary and tertiary prevention. More primary research needs to be done in these countries to determine what strategies and interventions exist and the applicability of HIC interventions in LMICs.

## Background

Neurotrauma is a major global health problem [1, 2]. Current studies estimate a worldwide annual incidence in the range of 500–800 per 100,000 population per year [3]. At present, neurotrauma accounts for about 11.8% of total global disability-adjusted life years and it is estimated to become the 2nd leading cause of premature death and disability globally by 2020 [3–5].

Although neurotrauma typically refers to the injury to the brain and/or spinal cord, for this review, neurotrauma or traumatic brain injury (TBI), will focus on injuries to the head alone.

Road traffic collisions (RTCs) are one of the most common causes of neurotrauma [5–7]. This is especially so in low- and middle-income countries (LMICs) due to rapid urbanisation and motorisation without accompanying safeguarding measures [7–9]. For this review, RTCs will be defined as a collision or incident involving at least one motorised or unmotorised (i.e. pedestrian, cyclist) vehicle in motion, on a road to which the public has right of access [10]. For this review also, the World Bank economic classification is used to define countries as low- and middle-income and high income, where the former refers to any countries in the low, lower-middle and upper-middle-income groups [11].

Irrespective of the cause, this ‘silent epidemic’ poses a myriad of consequences ranging from the economic burden, burden to healthcare systems, major psychological, social and community impact, as well as demographic impact, as the burden tends to fall disproportionately on children and young adults [9, 12–14].

The high individual, societal and global implications of neurotrauma indicate that it is imperative to have preventative measures in place in order to lower morbidity and mortality [7]. These are not only limited to approaches which target injury occurrence (primary prevention) but also involve providing adequate medical response to manage and minimise harm following an injury (secondary prevention) and mitigating the sequelae and reducing consequent disability (tertiary prevention) [14–16]. These can be applied at societal, community, household and individual levels [14, 17].

In high-income countries (HICs), these strategies have been implemented in various forms which range from adapting the environment, legislation, safety education and skills training, to strengthening post-trauma response systems and improving access to acute and post-acute care [14, 18–21]. However, LMICs often lag far behind in this area despite the higher toll of neurotrauma in these societies [4, 6, 22]. It is also clear that not all HIC approaches may be applicable to the LMIC context due to differences in environment, resources, population, culture and infrastructure [23, 24].

To date, several reviews have been carried out to identify, examine, and study the effectiveness of specific preventative approaches in particular regions or countries [7, 23, 25–36]. A previous scoping review has reported on interventions to reduce road traffic injuries, but this was limited to the African continent, and another review has specifically focused on physiotherapy after neurotrauma [37, 38].

Given the disparity between LMICs and HICs, the purpose of this scoping review was to provide an evidence map of the different strategies and interventions for neurotrauma and RTCs prevention that are available in both contexts. Examining the extent of research in this area will help identify gaps in the current literature, and potentially influence policy and practice relating to neurotrauma and RTCs prevention globally.

The objective of this scoping review is to identify and quantify the breadth of evidence on strategies and interventions in neurotrauma prevention, provide a descriptive overview of what these are, where they are implemented, and ascertain contextual factors that influence their implementation.

## Methods

This scoping review is reported in accordance with the PRISMA Extension for Scoping Reviews (PRISMA-ScR) Checklist [39] (see Additional file 1) and guided by a detailed protocol that was registered with Open Science Framework on 5th April 2019 (https://osf.io/s4zk3/). This protocol was also recently published in BMJ Open [40].

This scoping review is informed by a methodological framework for conducting scoping reviews proposed by Arksey and O’Malley (2005) [41]. The following five stages were included:

(1) Identifying the research question, (2) Identifying relevant studies, (3) Study selection, (4) Charting the data and (5) Collating, summarising and reporting the results.

### Research questions

The overarching review question was: “What are the global strategies and interventions in neurotrauma and RTCs prevention?” The sub-review questions are as follows:

1. What are the strategies and interventions in neurotrauma and RTCs prevention in LMICs?
2. What are the strategies and interventions in neurotrauma and RTCs prevention in HICs?
3. In what settings are these strategies and interventions carried out (i.e. school-based/community-based)?
4. What are the contextual factors that can affect or influence the implementation of these strategies and interventions?

### Eligibility criteria

As a result of time restrictions and cost of translation services, only publications in English were included. Owing to the call for global awareness on the prevention of road traffic collisions at the 27th World Health Assembly, this review included papers published since 1974. In order to cover a wide spectrum of literature, there was no restriction as to the types of studies included in the review.

The other eligibility criteria are given below, which follows the Population, Concept and Context (PCC) mnemonic [42].

#### Participants

The review included interventions targeted at adults and children, where children are defined as those below the age of 18 years. These could be road users, road traffic collision victims, neurotrauma patients, or those providing care or assistance for neurotrauma patients or road traffic collision victims.

#### Concept

Any strategies and interventions implemented for the prevention of neurotrauma or RTCs were included in this review. These encompassed primary prevention—referring to measures that eliminate the occurrence of RTCs or neurotrauma; secondary prevention—which are any interventions or strategies that form part of the pre-hospital care system; and tertiary prevention—which are any form of rehabilitative strategies and interventions for neurotrauma patients. For RTCs, the review included strategies and interventions that prevent collisions, and prevent neurotrauma should a collision occur. Only established and context-specific interventions, with or without reported outcomes, were included.

#### Context

The strategies and interventions were carried out or delivered in any LMIC or HIC. Papers involving multiple contexts were also eligible for inclusion.

### Search strategy

The search strategy for this review was finalised after consultations with an academic librarian, exploratory searches using the key concepts ‘neurotrauma’, ‘road traffic collisions’, ‘prevention’ and their synonyms, and piloting in one database (see Additional file 2). The strategy was used to search the following international electronic databases; MEDLINE (Ovid, 1946-present), Excerpta Medica, EMBASE (Ovid, 1947-present), Cumulative Index to Nursing and Allied Health Literature, CINAHL (EBSCO host, 1984-present), Global Health (EBSCO host, 1973-present) and Cochrane Database of Systematic Reviews (1996-present). The databases were selected so as to allow for a good coverage of primary and secondary publications with a multidisciplinary, neurosurgical and global health focus. Searches on these databases were conducted between 5th and 10th April 2019.

“Grey” literature was searched either manually or using a combination of keywords in grey literature databases and non-government websites with a focus on neurotrauma and road safety. Details of these databases and websites can be found in Additional file 3. Manual searching was also carried out in websites of transport ministries and road traffic safety authorities of the following countries: India, Colombia, Pakistan, Tanzania, Zambia, Ethiopia, South Africa, Nigeria, Myanmar, Indonesia and Malaysia. These countries were selected as they are the collaborating countries in the NIHR Global Health Research Group for Neurotrauma [43].

Due to the surplus of original hits, bibliographic searching was carried out using the reference list of only two articles which are reviews on the prevention of road traffic injuries [7, 37]. These articles were also used for citation searching using Google Scholar.

Searching for additional sources was completed on 29th May 2019.

### Citation management and study selection

All articles retrieved from the database searches were exported and stored in EndNote X7 bibliographic and reference manager.

Post-deduplication, a two-stage screening process was carried out, where titles and abstracts were screened independently by three reviewers (SMS, MMK and DMS), followed by full-text screening of publications which were deemed eligible (‘include’), and those where the title or abstract did not provide sufficient information on eligibility (‘uncertain’). Any uncertainty about study selection was resolved through consensus and re-examination of eligibility criteria.

### Data extraction, collation and summary

A customised data extraction form was developed by two reviewers (SMS and DMS) using Microsoft® Word for manual data extraction, informed by relevant methodological guidance [44]. This was piloted on 10 randomly selected papers, updated iteratively and used for the remaining studies. Extracted data included: first author, publication year, publication type, country of study, study aims/purpose, study design, study population, intervention and intervention details, setting, outcomes and key findings which included any effects and contextual factors.

The following strategies were used to present the results of this review: (1) a Preferred Reporting Items for Systematic Reviews and Meta-Analysis (PRISMA) flow diagram to present the study selection process; (2) tables and figures to present data extracted from the eligible papers; (3) a narrative summary describing the studies in relation to the objective and review questions. As there were a large number of eligible articles, these were divided into primary studies and reviews, and categorised based on the level of prevention.

## Results

### Descriptive numerical summary

**Fig. 1:**
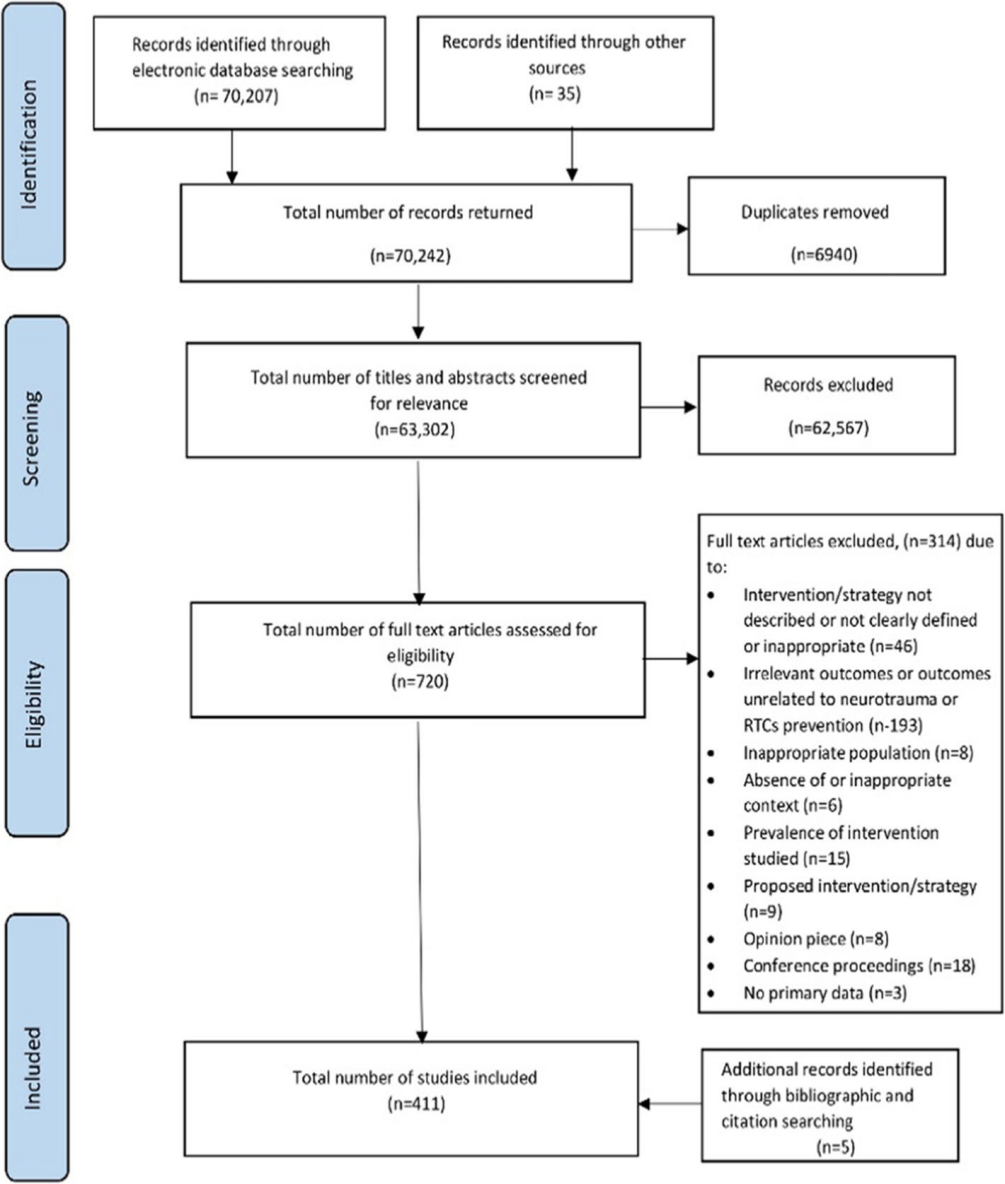
PRISMA flowchart of the study selection process

The electronic searches yielded 70,242 potentially eligible citations. After de-duplication, 63,302 citations underwent title and abstract screening, leaving 720 articles that were reviewed for eligibility. After full-text review, 406 articles were retained. Bibliographic and citation searching resulted in 5 additional studies being identified, with a final number of 411 studies being included in the review. The flowchart showing the selection process from identification to final inclusion is depicted in Fig. 1.

### General characteristics of included studies

Of the 411 included studies, 349 were primary studies and 62 were reviews. The key characteristics of both types of studies are summarised in Tables 1 and 2.

**Table 1:** Key characteristics of included studies: primary studies (*n* = 349)

Criterion	Characteristic	Number of studies (%)
Study design/publication type	Experimental	123 (35.2)
	Observational	116 (33.2)
	Descriptive	25 (7.1)
	Discussion paper/report	58 (16.6)
	Qualitative	19 (5.4)
	Mixed-methods	8 (2.5)
Country of origin of study	LMIC	65 (18.6)
	HIC	284 (81.4)
Intervention type	Primary prevention	249 (71.3)
	Secondary prevention	57 (16.3)
	Tertiary prevention	40 (11.5)
	Multiple levels of prevention	3 (0.9)
Year of publication	1974–1983	3 (0.9)
	1984–1993	24 (6.9)
	1994–2003	51 (14.6)
	2004–2013	114 (32.7)
	2014–2019	157 (44.9)
Population	Adults only	68 (19.5)
	Children only	72 (20.6)
	Adults and children	209 (59.6)
Setting	National	76 (21.8)
	State/province	114 (32.7)
	City/town/village	84 (24.1)
	Neighbourhood/home/school/health facility/workplace/community	59 (16.8)
	Not reported	16 (4.6)

**Table 2:** Key characteristics of included studies: reviews (*n* = 62)

Criterion	Characteristic	Number of studies (%)
Review type	Systematic review	37 (59.7)
	Meta-analysis	8 (12.9)
	Literature review	15 (24.2)
	Scoping review	2 (3.2)
Country of origin of included primary studies	LMIC	5 (8.1)
	HIC	42 (67.7)
	LMIC and HIC	15 (24.2)
Intervention type	Primary prevention	41 (66.1)
	Secondary prevention	3 (4.8)
	Tertiary prevention	18 (29.1)
Year of publication	1974–1983	–
	1984–1993	–
	1994–2003	8 (12.9)
	2004–2013	22 (35.5)
	2014–2019	32 (51.6)
Population	Adults only	9 (14.5)
	Children only	5 (8.1)
	Adults and children	48 (77.4)
Setting	National	8 (12.9)
	State/province	1 (1.7)
	City/town/village	10 (16.1)
	Neighbourhood/home/school/health facility/workplace/community	11 (17.7)
	Multiple	5 (8.1)
	Not reported	27 (43.5)

### Primary studies

**Fig. 2:**
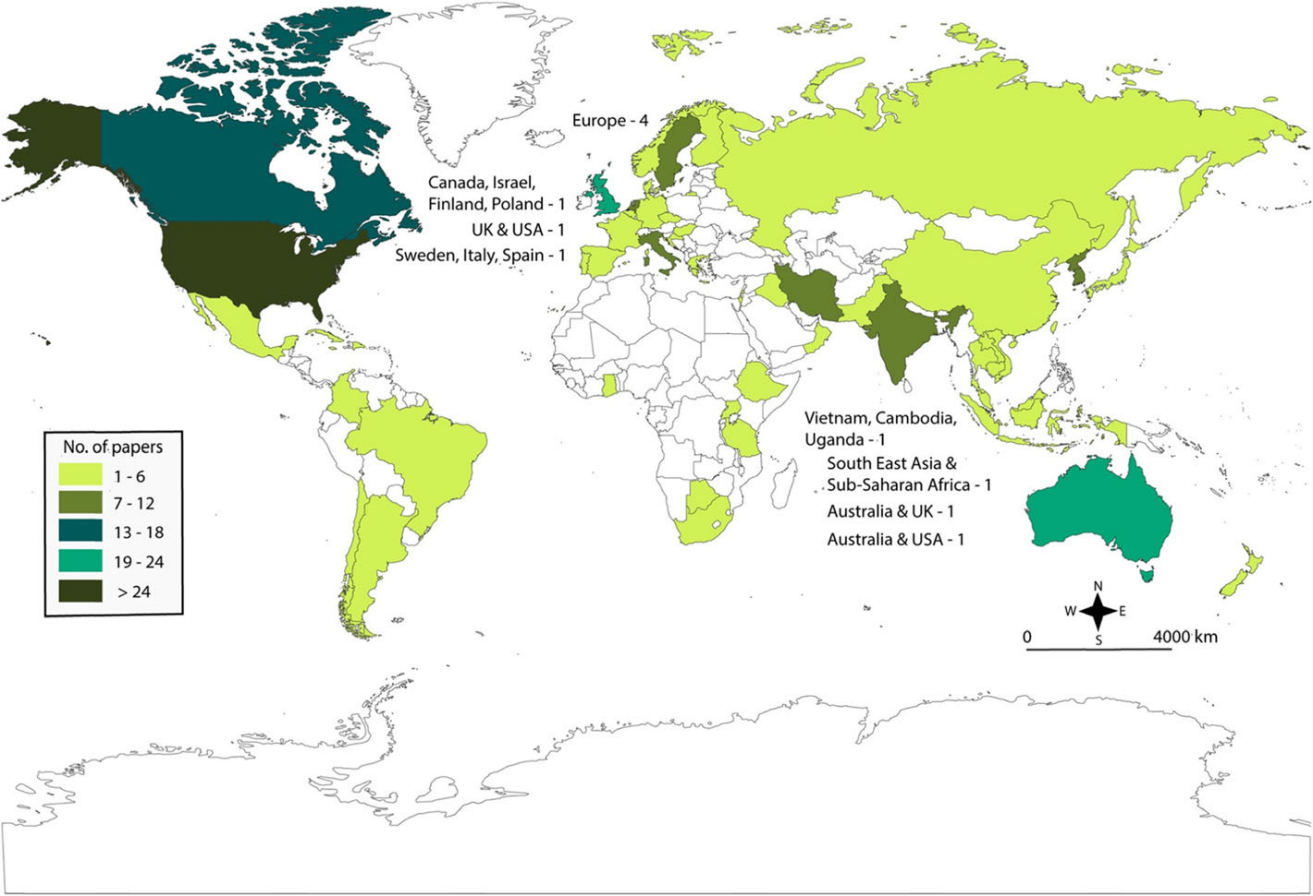
Map of the location of where strategies and interventions from included studies were implemented

The majority of the primary studies were from HICs, with less than 20% of studies from LMICs. Figure 2 shows a map of the distribution of studies, where many came from three countries—United States of America (USA), United Kingdom (UK) and Australia. Most LMICs gave rise to 6 or fewer publications.

**Fig. 3:**
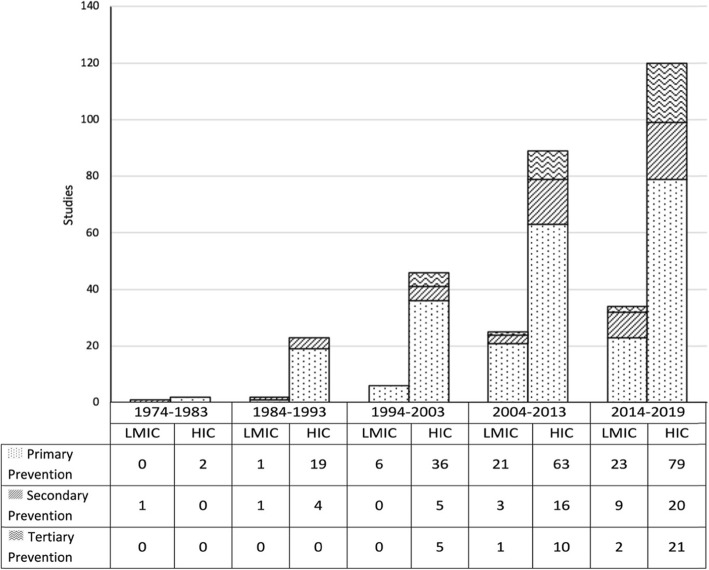
Distribution of strategies and interventions by types in HICs and LMICs over time (1974–2019)

Figure 3 depicts the distribution of interventions and strategies over time in both HICs and LMICs. Most of the studies emerged after the 1990s, both in HICs and LMICs, focussing on primary prevention of neurotrauma, which accounted for nearly three-quarters of the primary studies.

### Reviews

For the reviews, nearly three-quarters (72.6%) were systematic reviews and/or meta-analyses. Most reviews were published after 2000. Primary preventative interventions and strategies were reviewed in 41 papers (66.1%), 18 reviewed tertiary (29%) and only three papers reviewed secondary approaches.

Most of the primary papers in the reviews came from HICs (67.7%) with only 5 reviews from LMICs alone. Fifteen reviews (24.1%) included papers from both HICs and LMICs. The majority of the reviews described interventions targeting adults and children and did not report the setting.

### Strategies and interventions

#### Primary studies

A total of 349 papers were included in this review. The individual strategies and interventions in each category for primary and secondary prevention with the accompanying publication information can be found in Additional file 4.

##### Primary prevention

A total of 252 papers described primary preventative strategies and interventions. All these are categorised and summarised in Table 3. Some studies are included more than once within the results as they discussed more than one strategy or intervention.

**Table 3:** Summary of interventions and strategies for primary prevention of neurotrauma from primary studies (*n* = 252)

Intervention/strategy type	Personal safety/protective equipment	Education/training/awareness-raising	Legislation/policy	Enforcement	Engineering	Multi-component
No of studies	58	48	96	36	51	4
Country type						
LMIC	14	15	17	9	12	1
HIC	44	33	79	27	39	3
Study design/publication type						
Experimental	2	16	49	20	14	2
Observational	28	3	23	5	15	1
Descriptive	13	3	9	–	1	–
Discussion paper/report	4	20	14	10	16	1
Qualitative	7	3	1	1	4	–
Mixed methods	4	3	–	–	1	–
Setting						
National	11	12	34	15	20	1
State/province	15	6	47	10	8	–
City/town/village	27	12	10	10	18	2
Neighbourhood/school-based/health facility-based/workplace	2	13	4	–	2	1
Not reported	2	5	1	1	3	–
Years						
1974–1983	1	–	–	–	–	–
1984–1993	6	3	7	1	5	–
1994–2003	11	7	23	2	4	2
2004–2013	11	21	31	16	21	1
2014–2019	29	17	35	17	21	1
Population						
Adults and children	34	23	49	32	43	3
Adults only	6	15	13	4	6	–
Children only	18	10	34	–	2	1

- Personal safety/protective equipment

Fifty-eight papers explored the use of various personal safety or protective equipment against neurotrauma or road traffic collisions. Three-quarters of the studies came from HICs. Most of the papers were on helmet use and were from both HICs and LMICs. Although most of the studies discussed helmets in relation to road safety, some examined their use in sports, combat and for work safety. All these non-road safety papers on helmet use were from HICs.

Similarly, seat belt use, child car restraints and conspicuity equipment (e.g. high visibility jackets or tapes) were also described in both HIC and LMIC papers. One HIC study discussed other protective sports equipment other than helmets [45].

The setting for this strategy ranged from cities and rural areas to state and nationwide.

- Education/training/awareness-raising

This was described in 48 papers, the majority of which were from HICs. The most common type was road safety education or training for various road users. This included driver or motorcycle rider education or training, and pedestrian safety education. One study from an LMIC described a peer education programme where workers were educated on road safety to be road safety ambassadors in industrial and community settings [46]. Two HIC studies described the education of parents on issues surrounding child safety seat use [47, 48]. As for other causes of neurotrauma, five HIC studies explored the education of parents and nurses on abusive head trauma in infants and young children [49–53].

For sports injuries, two studies, one from an LMIC and the other from a HIC, discussed training and education of athletes and coaches to reduce neurotrauma from rugby and football respectively [54, 55].

Materials and methods used for education or training included lectures, demonstrations, videos/DVDs, simulation, quizzes, mobile applications, manuals and worksheets. Another common approach, found predominantly in HICs was the use of campaigns. Most of these campaigns involved media activities to raise awareness about road safety. Two of these were organised at a school-level, both from HICs. Three studies from HICs also discussed campaigns to raise awareness on head trauma in infants as a consequence of abuse.

Most of the interventions took place within the community, both for HICs and LMICs, except for media campaigns which were usually carried out at a national or state level.

- Legislation/policy

There were 96 papers that studied different legislation and policies for the prevention of RTCs and neurotrauma. Over 80% of papers were from HICs, where more than half discussed graduated driver licensing system (GDLS) and helmet laws or policies (GDLS is a system designed to allow new drivers to develop their driving skills and experience in well-defined stages [56]). Although the majority of the LMIC studies were also on the helmet laws, only two looked at GDLS [56, 57]. Other policies and legislation found in both LMICs and HICs were on seatbelt and child passenger safety, drink-driving, speeding, cell-phone or texting bans and general road safety.

HIC studies assessed policies and legislation surrounding vehicle roadworthiness, road safety at work, road safety audit, fitness to drive and licensing restrictions, congestion charging schemes, traffic signs or symbols, crossing guards, vehicle and road user conspicuity and rewards for safe driving or reporting unsafe driving.

Most of these strategies were implemented either at a national or state-wide level, although some were carried out at the workplace.

- Enforcement

Thirty-five references examined enforcement strategies and interventions, with only 9 from LMICs. These LMIC studies focussed on traffic policing or patrolling and enforcement of traffic laws, as well as on penalty systems for errant road users. Similar interventions were described in studies from HICs, but the majority described photo enforcement programmes through speed cameras and red light running cameras. One study looked at a school bus stop-arm camera, which cited drivers who would illegally pass a stopped school bus [58]. Both HIC and LMIC studies also discussed enforcement of drink and drug-driving and enforcement of laws on the use of helmets, seat belts and child car safety seats.

Given the nature of the interventions or strategies, most were carried out at national or state-level, or within cities or towns.

- Engineering

Out of the 51 papers in this category, more than three-quarters came from HICs and discussed two approaches: road engineering and vehicle engineering. Most of the studies on road engineering from both HICs and LMICs described various road modifications including installation of roundabouts, changing road curvature, speed modification and other traffic calming measures. Both HIC and LMIC papers also described other interventions such as traffic and pedestrian countdown signals and exclusive lanes for bicycles, motorcycles and buses. Only HIC studies discussed audio-tactile lane-markings and street lighting. One LMIC study explored the use of pedestrian footbridges [59].

Vehicle engineering approaches were examined in mostly HIC studies. These included vehicle design, in-vehicle technologies such as seat belt reminders and airbags, as well as collision avoidance technologies such as anti-lock braking systems, alcohol ignition interlocks, intelligent speed adaptation, blind-spot monitoring and lane departure warning systems. The three LMIC studies in this category discussed collision avoidance measures through anti-lock braking systems, brake checks for bicycles, and motorcycle roadworthiness with installation of horns and other warning devices [56, 60, 61].

The settings for these interventions varied, although these were largely implemented in cities or towns, as well as at a national level.

- Multi-component

Four studies discussed interventions or strategies with multiple components where three of the papers came from HICs. Each strategy had different combinations of the various approaches described above, with a unified focus on education and legislation or policies. The HIC strategies were carried out either in the community or in cities, whereas the LMIC strategy was implemented nationally.

##### Secondary prevention

Secondary preventative strategies and interventions were discussed in 60 papers. The individual approaches are categorised and summarised in Table 4.

**Table 4:** Summary of interventions and strategies for secondary prevention of neurotrauma from primary studies (*n* = 60)

Intervention/strategy	Pre-hospital airway management	Pre-hospital fluid resuscitation	Pre-hospital triage/imaging	Ambulance services	Air EMS	Physician-staffed/physician-led EMS	Pre-hospital care training	Direct transport to neuro-surgical centre/theatre	Organised trauma/emergency medical system	Lay first responders	Crash notification and response systems	Multiple interventions
No. of studies	11	1	3	8	10	7	7	1	4	2	3	3
Country type												
LMIC	–	–	–	5	1	1	4	–	2	1	–	–
HIC	11	1	3	3	9	6	3	1	2	1	3	3
Study design/publication type												
Experimental	3	–	–	–	–	–	–	1	2	–	1	–
Observational	7	1	2	4	9	4	1	–	2	–	–	–
Descriptive		–	–	1		1	1	–	–	1	1	–
Discussion paper/report	1	–	1	2	1	2	4	–	–	1	1	3
Qualitative	–	–	–	1	–	–	1	–	–	–	–	–
Mixed methods	–	–	–	–	–	–	–	–	–	–	–	–
Setting												
National	2			4	1	1	2		1	1	1	–
State/province/district/county	6	–	1	2	4	4	2	1	3	1	1	1
City/town/village	3	1	2	1	3	1	1	–	–	–	1	–
Neighbourhood/school-based/health facility-based/workplace	–	–	–	–	1	1	2	–	–	–	–	–
Not reported	–	–	–	1	1	–	–	–	–	–	–	2
Years												
1974–1983	–	–	–	–	–	1	–	–	–	–	–	–
1984–1993	1	–	–	1	1	1	–	–	–	1	–	–
1994–2003	2	–	–	1	–	–	1	–	–	–	1	–
2004–2013	6	–	1	–	3	2	2	–	3	1	1	1
2014–2019	2	1	2	6	6	3	4	1	1		1	2
Population												
Adults and children	9	1	3	7	8	7		1	4	1	3	3
Adults only	1	–	–	–	1	–	7	–	–	–	–	–
Children only	1	–	–	1	1	–	–	–	–	1	–	–

Note: *EMS* emergency medical services

The majority of the papers came from HICs and explored various forms of post-collision pre-hospital care, most of which were on airway management through intubation and ventilation. Other HIC papers looked at pre-hospital triage including one which described the use of a mobile stroke unit for imaging in neurotrauma and pre-hospital fluid resuscitation [62, 63]. Another HIC paper was on the direct transport of victims to neurosurgical centres or operating theatres, bypassing nearby hospitals or health facilities [64].

Interventions reported in both LMICs and HICs included emergency medical services (EMS). The HIC papers mostly described Air EMS where either a helicopter or aircraft was used in the transport of RTC or neurotrauma victims. The other HIC papers discussed a physician-led or physician-staffed EMS where a trained physician would attend the scene of trauma either together with or separate from other EMS staff. Most LMIC papers were on ground EMS or ambulance services.

Three papers, all from HICs, described different forms of collision notification and response systems that enable the occurrence of a collision or trauma to be reported or identified swiftly, and allow for EMS to arrive rapidly at the scene [65–67].

Organised trauma systems were explored in four papers, 2 from HICs and 2 from LMICs. The LMIC systems focussed on life support (Basic Life Support by first responders and Advanced Life Support by paramedics), whereas the HIC systems included triage, transport and a multidisciplinary pre-hospital management of patients [68–71].

Out of the 7 papers on pre-hospital care training, the majority were from LMICs, where training was not only for healthcare staff but also for lay responders, namely the police and public transport providers. There were also two papers describing the role of lay responders in providing first aid to RTC or neurotrauma victims, where the HIC paper discussed the role of police, and the LMIC paper, taxi drivers [72, 73].

Three papers were on multiple interventions, which described a combination of pre-hospital services, triage and resuscitation. All papers were from HICs.

The majority of the interventions or strategies were carried out at a state or province-wide level. Some were implemented nationally, while others occurred in cities or towns.

##### Tertiary prevention

Forty papers dealt with various rehabilitative strategies and interventions for neurotrauma patients and are summarised in Table 5. The description of these interventions can be found in Additional file 6.

**Table 5:** Summary of intervention and strategies for tertiary prevention of neurotrauma from primary studies (*n* = 40)

High-income countries							
First author	Year	Country	Study design/publication type	Intervention/strategy	Population	Setting	Provider
Bay	2019	USA	Experimental	Mindfulness-based group therapy	Adults with mild to moderate TBI	Rehabilitation centre	Trained facilitators with mental health and/or mindfulness backgrounds
Bedard	2014	Canada	Experimental	Mindfulness-based cognitive therapy	Adults with a history of TBI	Community sites in three cities	Clinicians
Bombadier	2009	USA	Experimental	Telephone contact with patient- counselling, brief motivational interviewing and education	Adults and children with TBI	Patient’s home	Rehabilitation staff
Brett	1998	USA	Experimental	Cognitive rehabilitation	Students with TBI (from Grade 9-12)	Inter-city public school	Teacher
Caglio	2012	Italy	Descriptive	3D video game with different navigational tasks	24-year-old man with TBI	Rehabilitation centre	Neuropsychologist
Cantor	2014	USA	Experimental	STEP: Short-Term Executive Plus (a cognitive rehabilitation programme)	Adults with a history of TBI	Medical centre	Licensed psychologists, psychology post-doctoral fellows
Chua	1999	Singapore	Observational	Multidisciplinary rehabilitation	Adults with TBI	Hospital	Rehabilitation team
Combs	2018	USA	Mixed-methods	Yoga and mindfulness	Adults with TBI	Veteran’s Affairs health care centres	Psychology post-doctoral fellow and Registered Yoga Teacher
Connor	2016	USA	Descriptive	Computer games and meta-cognitive training	Adults with TBI	Neurorehabilitation department	Speech therapist
Cooper	2017	USA	Experimental	Computerised cognitive rehabilitation, traditional cognitive rehabilitation and integrated cognitive rehabilitation	Adults service members with mild TBI	TBI clinic at Army Medical centre	Speech language pathologists, occupational therapists, doctoral psychologists, clinic staff
De Luca	2019	Italy	Descriptive	Conventional cognitive therapy in a computer-assisted rehabilitation environment	15-year-old adolescent with severe TBI	Rehabilitation centre	Skilled neuropsychologist
Evald	2015	Denmark	Descriptive	Smartphone	Adults with TBI	City	Neuropsychologist
Fogelman	2012	USA	Discussion paper/report	Exercise therapy	Patients with mild TBI	Not reported	Not reported
Gabbatore	2015	Italy	Experimental	Cognitive Pragmatic Treatment	Adult patients with TBI	Rehabilitation centre	Psychologist
Gardiner	2015	USA	Experimental	Neurologic Music therapy (NMT)	Military veterans who had been diagnosed with TBI	Veterans health centre	Musician who is a licensed psychologist, board-certified neuropsychologist trained in NMT
Goldshtrom	2010	USA	Descriptive	Rhythmex: Rhythmic exercises with auditory cues	24-year-old female with TBI	Physical therapy clinic	Physical therapist
Hoosan	2010	UK	Qualitative	Return to work rehabilitation	Adults with TBI	Brain injury service clinic	Occupational therapists
Keegan	2019	Australia	Experimental	INSIGHT: Improving Natural Social Interaction: Group reHabilitation after Traumatic Brain Injury	Adults with TBI	University clinic and other community settings	Speech-language pathologist
Kline	2016	USA	Discussion paper/report	Art therapy	Any survivor of TBI	Rehabilitation facility	Not reported
Linton	2018	USA	Experimental	Trabajadora de salud- solution focused brief therapy	Adults with TBI	Patient’s home	Lay health workers
Nayak	2000	USA	Experimental	Music therapy	Patients with history of moderate to severe TBI	Rehabilitation institute	Music therapist
Nelson	2013	USA	Experimental	Interactive metronome therapy	Soldiers with mild to moderate TBI	Medical centre	Neuropsychologist
Salazar	2000	USA	Experimental	In-hospital cognitive rehabilitation programme	Military personnel with moderate-to-severe closed head injury	Hospital	Board-certified psychiatrist, certified occupational therapist, speech pathologist and one rehab assistants
Saux	2014	Argentina	Descriptive	Cognitive rehabilitation	Adults with TBI	Hospital	Neuropsychologist
Scheenen	2017	The Netherlands	Descriptive	UPFRONT-preventive Cognitive Behavioural Therapy	Adults with mild TBI	Medical centre	Experienced healthcare psychologist
Seguin	2018	Canada	Experimental	Ready! Set? Let’s Train!: a type of cognitive rehabilitation	Children aged 10–17 years	School or home	Neuropsychologist
Serino	2007	Italy	Experimental	Working Memory training	Adults with TBI	Rehabilitation Centre	Neuropsychologist
Solana	2014	Spain	Experimental	Intelligent assistant therapy	Adults with TBI	Hospital	–
Sullivan	2014	Australia	Qualitative	Real-life activities in rehabilitation	Young men with TBI	Home	Not reported
Swaine	2000	Canada	Experimental	Coordinated multidisciplinary rehabilitation	Children with TBI	Children’s Hospital	Multidisciplinary team
Thaut	2009	USA	Experimental	Neurologic music therapy	Adults with TBI	Not reported	Board-certified musical therapist
Tiwari	2018	USA	Descriptive	Home-based circuity training	17-year-old with TBI	Home	School physical therapist
Twamley	2014	USA	Experimental	CogSMART: Cognitive Symptom Management and Rehabilitation Therapy	Veterans with history of TBI	Rehabilitation clinic	Not reported
Van Spanje	2019	The Netherlands	Observational	The Brainz programme: Cognitive and physical rehabilitation	Adults with TBI	Home and rehabilitation centre	Cognitive trainer, pscyhomotor trainer or physiotherapist, family
Vik	2018	Norway	Experimental	Music training	Adults with mild TBI	Rehabilitation centre	Music instructor
Wood	2011	UK	Experimental	Implementation intentions and Goal Management training	Adults with TBI	Rehabilitation centre	Doctoral student in clinical psychology
Yost	2013	USA	Qualitative	Qigong	Service members with TBI	Rehabilitation centre	Qigong master
**Low- and middle-income countries**							
Fernandez	2012	Cuba	Experimental	RehaCom: software for computer-assisted cognitive rehabilitation	Adult patients with TBI	Rehabilitation centre	Neuropsychologists
Hedge	2014	India	Discussion paper/report	Music-based cognitive remediation therapy	All patients with TBI	Not reported	Not reported
Soeker	2017	South Africa	Experimental	MOOSE: Model of Occupational Self-Efficacy	Adults with mild to moderate TBI	Occupational therapy department	Occupational therapist

Note: *USA* United States of America, *TBI* traumatic brain injury, *3D* three dimensional, *UK* United Kingdom

More than 90% of the papers were from HICs where the majority discussed rehabilitation of cognitive function through various approaches including the use of video or computer games, virtual reality systems, music therapy and electronic devices including mobile phones. Two studies investigated the role of mindfulness in improving cognitive functioning, where one also assessed physical and emotional functioning post-therapy [74, 75]. These elements were also examined in yet another study on mindfulness, which included yoga as a co-intervention [76]. All these papers were from HICs.

Similarly, other HIC studies discussed the role of various interventions or strategies in improving different aspects of functioning in neurotrauma patients. These ranged from art therapy, music therapy, rhythmic exercises with auditory cues and qigong. Two studies looked at multidisciplinary rehabilitation programmes which involved various healthcare workers with different expertise including physiotherapy and speech therapy [77, 78].

Strategies and interventions that addressed only emotional rehabilitation were described in three HIC studies and included cognitive behavioural therapy, telephone counselling and a form of psychotherapy carried out by lay workers [79–81]. Physical rehabilitation was discussed in two HIC studies using exercise therapy and home-based circuitry training [82, 83].

Occupational rehabilitation was explored in two papers, one using real-life activities as part of rehabilitation. Both papers were from HICs [84, 85].

Only three papers came from LMICs and described computerised cognitive rehabilitation, cognitive music therapy and occupational therapy [86–88].

Most of the interventions were carried out in rehabilitation centres, hospitals or medical centres. A few were carried out in schools, where the target population were children, and some in community centres or patient’s homes.

#### Reviews

Sixty-two reviews were included. The strategies and interventions together with the publication information have been categorised and can be found in Additional file 5. The description of the reviewed tertiary interventions is also found in Additional file 6.

##### Primary prevention

This was explored in 41 reviews where most included primary papers from HICs and assessed single interventions. The strategies and interventions that were most studied related to enforcement and legislation or policy. The three reviews that had primary papers only from LMICs evaluated multiple strategies and interventions.

##### Secondary prevention

Only three reviews discussed secondary prevention. Two included HIC papers, which looked at pre-hospital tracheal intubation and direct transport to a neurotrauma centre, and one included LMIC papers, which assessed trauma systems [31, 89, 90].

##### Tertiary prevention

Eighteen papers evaluated different rehabilitative interventions and strategies. The majority of reviews included primary papers from HICs or a combination of HICs and LMICs. Only one review, on the use of acupuncture, included primary papers from China alone [91].

#### Contextual factors

This was discussed in 58 of the primary studies and four of the reviews and varied depending on the studied intervention or strategy. These are summarised based on the interventions described and are given below.

##### Primary prevention

- Personal safety/protective equipment

Twenty-two papers looked into contextual issues relating to personal safety or protective equipment. Thirteen were on helmets, four on seatbelts, four on child safety seats and one on both helmets and seatbelts.

The use of personal safety equipment was influenced by attitude and knowledge of the protective effects as well as awareness of laws or campaigns promoting use. Relating to that, the existence of laws or enforcement determined whether such equipment was used or otherwise, especially for short-distance travel [92]. External pressure played a role, especially in children, where peers and family influenced if an individual would own or use such equipment. Likewise, promotional activities by healthcare professionals or the media also seemed to affect individual use. The history of an accident or head injury was found to increase use, where the danger of non-use had been real to the individual or their loved ones. All these were seen in both HIC and LMIC papers.

Cost and availability also affected whether safety equipment was utilised, especially in LMICs. In the HIC studies on helmets, the type and fit were also linked not only to use but whether the helmet was effective in protecting against head injury. In one LMIC study on child car seats, the make of the car was sometimes a barrier to use due to a poor fit [93].

- Education/training/awareness-raising

Contextual issues about education and awareness were discussed in nine studies. Four studies were on campaigns, all from HICs, where factors affecting implementation were linked to the methods used. Successful campaigns were those that were largely interactive, offered simple explanations and used multiple channels to deliver the message. The location of campaigns also played a role, where success was greater when it was carried out in areas with the highest risk.

Resource also affects the success of educational ventures, where inexpensive methods are likely to be adopted by others and are more sustainable, and methods requiring less manpower would have the capacity to cover a larger target population.

However, personal and environmental factors can result in a lack of change in attitude or behaviour despite the acquisition of knowledge. For example, in a study on a driving course for mature drivers, perceptions that it is just a ‘refresher’ or attending just to please family members resulted in a lack of change in driving behaviour [94]. Similarly, a Graduated Driving programme designed to promote safe driving among teenagers could not ameliorate risky driving due to peer influence [95].

Local contexts are also important for the success of any educational programmes, where this should be carried out in the language most familiar to participants, and take into account local conditions and programmes, as discussed in one LMIC paper [46].

- Legislation and enforcement

Six studies discussed contextual issues concerning enforcement and legislation. In many LMICs, legislation with inconsistent and inadequate enforcement was found to be a barrier to the success of the strategy or intervention. Also, manpower and resource management are important factors that allow for enforcement to be carried out efficiently and successfully, as described in both HIC and LMIC papers.

Two HIC studies examined traffic signs, where factors influencing their successful implementation were linked to design, visibility, knowledge and universal standardisation [96, 97].

Public knowledge of and attitude towards legislation and enforcement also play a role in the success of such strategies and interventions, for example, perceiving road laws as coercive, non-beneficial or for instilling fear can be a barrier to road safety, as explored in one LMIC study [98].

- Engineering

Contextual factors pertaining to engineering strategies and interventions were explored in nine studies. Most studies discussed issues surrounding road engineering. Despite road engineering programmes, poor design resulted in the structure or facility being non-protective and inconveniencing road users, especially pedestrians. In an LMIC study on pedestrian footbridges, good locations promoted use, although physical and psychological barriers resulted in non-use for some [59].

Another LMIC study on exclusive motorcycle lanes found this was highly accepted by motorcyclists as it facilitated riding and reduced commute time, in addition to promoting safety on the road [99].

A further three studies from HICs looked at vehicle engineering. One study from Spain showed that better car designs encouraged speeding [100]. Additionally, vehicle technologies, while helpful and promote safety, could also lead to a false sense of security, especially among novice drivers. Conversely, not all cars could be fitted with such technologies, or even with seatbelts, due to their age and make, which is an issue commonly seen in LMICs.

##### Secondary prevention

- Ground and Air EMS

In the five studies discussing contextual factors on ambulance services, issues such as cost, access and awareness of services affected the implementation of this strategy, especially in LMICs. Response time was also discussed where the presence of heavy traffic and lack of dedicated ambulance lanes resulted in the delay in scene arrival. This resulted in a preference for private transport, which was considered more convenient.

The success of the ambulance service also relied on the resources available, not only in terms of numbers of vehicles for dispatch but also in the number of dedicated and trained staff who would be able to resuscitate and monitor patients so the ambulance was not simply a transport medium. Again, this was described in the LMIC papers, where such resources were often lacking.

In the four HIC studies on air EMS, cost was also a factor that affected the success of this intervention. Unlike ground EMS, response time was swift for air EMS, as well as the ability to access remote areas. However, issues with street landing, especially when there is congestion, and space restriction within the helicopter made it difficult to carry out en-route resuscitation. Relating to that, the use of a larger aircraft, such as a Fokker 50, mitigated problems of space, noise and vibration.

- Lay responders and pre-hospital care training

Three studies described contextual factors in relation to lay responders and pre-hospital training. Two LMIC studies looked at first aid training of taxi drivers, where attitudes influenced the implementation of this intervention. One study showed a positive attitude among drivers where they found the training increased their confidence, whereas, in the other study, drivers felt that it was not necessary as their role is only for transport [73, 101]. Likewise in the HIC study on lay responders, police were trained in basic life support, but they also felt their role is policing and not to respond to medical emergencies.

- Physician-staffed/physician-led EMS

The HIC study discussed the Rendezvous system, where an Emergency Doctor is also alerted and dispatched to the incident site, in addition to an ambulance with medical personnel [102]. In this system, the doctor is involved in pre-hospital care but is able to work independently from the EMS and not have to go back-and-forth to the hospital, allowing them to respond quickly to emergencies and deal with more incidents.

##### Tertiary prevention

There were six studies exploring contextual issues for various different strategies and interventions, all from HICs.

The success of various approaches was very much dependent upon patient acceptability, where interventions that were engaging, easy to learn or use, and related to real-life activities were preferred. The involvement of different healthcare professionals and family members in rehabilitation also ensured patients received holistic care and were better adjusted when returning home or to work. Success was also influenced by the timing of the intervention or strategy where outcomes were better with rehabilitation being carried out not long after the injury.

## DISCUSSION

### Breadth of literature

This scoping review is the first review that has identified the breadth of strategies and interventions in neurotrauma and RTCs prevention globally. Most publications were from 2014, although there has been a consistent increase in published literature from 1990. The neurotrauma prevention literature mostly originated from HICs, in particular the USA, Australia, the UK and Canada. By contrast, there is still a paucity of original research literature from LMICs.

Most of the papers discussed primary prevention, followed by secondary prevention and tertiary prevention. Of these, 301 (74%) had a focus specifically on RTCs. Primary prevention concentrated on personal-level interventions such as the use of safety or protective equipment, and the provision of education or training; public-level interventions such as legislations and policies, law enforcement, and developing more robust road and/or vehicle engineering; and multi-component interventions which were a combination of both. Secondary prevention focused on pre-hospital care and various forms of EMS. Tertiary prevention encompassed a range of different rehabilitative strategies and interventions addressing mainly cognitive rehabilitation.

### Strategies and interventions in LMICs

Less than a fifth of publications came from LMICs alone. This is consistent with findings from other papers on the limited research on this topic from these countries [4, 6, 7, 25, 37].

All the primary preventative strategies and interventions in LMICs related to road safety. Most studies were in the legislation/policy category where the most common approach was the helmet policy. Other common strategies and interventions were helmet use, traffic calming and road modification and traffic policing or patrolling. The three reviews on LMIC papers described multiple interventions and strategies which included both personal and public-level approaches. These findings show that there are, indeed, a variety of strategies and interventions for the primary prevention of neurotrauma and RTCs in these countries. However, there was a clear deficiency in papers on vehicle engineering, particularly pertaining to in-vehicle safety technology. This is likely due to the fact that such technologies are costly, and also that many of the vehicles in these countries are dated, and unsuitable for this purpose [93].

For secondary prevention, most of the LMIC papers were on ambulance services and pre-hospital care training. While there were other strategies and interventions described, there was an absence of papers on pre-hospital care for neurotrauma or RTC victims. This could be explained by the fact that in LMICs, ambulances are used simply as a transportation medium, and there is a lack of dedicated and trained staff to provide pre-hospital care [103, 104]. Furthermore, well-defined trauma systems are lacking in LMICs, often due to resource constraints, resulting in the absence of pre-hospital care pathways and systems for managing trauma [105].

Tertiary prevention in LMICs included technological-based interventions, psychological interventions, occupational therapy, family-supported treatment, animal-assisted therapy, music therapy and acupuncture. Although the number of studies were few, the range of interventions show that LMICs have the capacity not only for high-cost approaches but also make use of pre-existing, non-resource constraining strategies for neurotrauma rehabilitation, such as acupuncture [91].

### Strategies and interventions in HICs

Primary prevention of neurotrauma and RTCs in HICs involved the utilisation of a diverse range of strategies and interventions that included both personal and public-level interventions, with most being under the legislation/policy category as well.

Only HICs had policies on vehicle safety and engineering which is in keeping with the fact that there was a greater use of technology in prevention in these countries, with studies on in-vehicle safety technology, vehicle design and collision avoidance technology. Another big difference was the greater emphasis on fitness to drive and licensing restrictions, where more HIC studies reported such policies, namely the GDLS. This corresponds to the fact that such licensing systems exist in more HICs than LMICs [106].

HIC papers described helmet use also in sports, combat and industrial and construction workers, in addition to its use in road safety. Some also included the use of other safety equipment to prevent head injury in sports. This shows that these activities are recognised in HICs as potential causes of neurotrauma and how measures need to be put in place to mitigate the consequence.

Another area identified as a cause of neurotrauma in HICs was non-accidental injury/infant abuse. There were papers on education and campaigns relating to the prevention of this phenomenon, notably in Canada and the USA.

The majority of the strategies and interventions in secondary prevention focused on pre-hospital care which would have been carried out either at the site of injury or within the EMS vehicle. There were also studies on physician-staffed or physician-led EMS and pre-hospital care training. This goes back to the way the trauma care system and pathways in HICs are designed to include not only life support but also a multidisciplinary management of patients and triage in the pre-hospital phase [68, 69]. In addition, some HIC trauma care pathways included direct transport to neurosurgical centres, bypassing nearer non-specialist centres [31, 64]. Again, this would be possible because of the availability of resources for pre-hospital resuscitation and care in HICs, where the patient would be stabilised and given preliminary non-surgical management before and during transport [64]. HICs also had more resources to have air EMS services, as seen by most of the papers coming from these countries.

Again, resource and technology played a role in prevention through collision notification systems and pre-hospital imaging, which were reported in only HIC papers.

Most of the tertiary prevention strategies and interventions were from HICs, which is especially reflected in the primary studies. Most addressed cognitive rehabilitation and utilised diverse approaches that ranged from technology- or healthcare centre-based interventions which were costly and labour-intensive to simple low-resource or community-based approaches, namely yoga and mindfulness.

Likewise, these strategies and interventions could also be used to rehabilitate other areas in the neurotrauma patient, particularly physical and emotional rehabilitation. Multidisciplinary rehabilitation was also described, where the patient would receive rehabilitation from a group of individuals with different expertise, and who would address different aspects of their functioning, in the same time period for rehabilitation [77].

An interesting finding was the use of mindfulness as both a primary and tertiary preventative approach [74–76, 107]. While the principals and conduct of the intervention were the same, the population and outcomes differed, where for primary prevention, mindfulness reduced distracted or dangerous driving in well-adults, whereas for tertiary prevention, it was used to address cognitive and emotional symptoms in neurotrauma patients.

### Settings of strategies and interventions

Owing to the fact that most of the papers were on primary prevention with a focus on legislation/policy, the settings were mostly at the national, state/province or city/town/village levels, regardless of context. It was interesting to note that some strategies and interventions were carried out at a national level in some countries, but at a state or city level in other countries, particularly in the legislation/policy category. This reflects the difference in the government system in countries, whether HICs or LMICs, where in some, each state or municipality is given autonomy to legislate on matters, which can sometimes result in a contradiction of State and National laws [108, 109].

Another interesting finding was in the ‘engineering’ category where most of the road engineering strategies and interventions were carried out in cities or towns, and vehicle engineering at a national level. This is not unexpected as road engineering is a safety approach that tends to be implemented by metropolitan governments whereas national governments tend to be involved in regulating standards for vehicles and safety mechanisms [109].

Educational interventions were more community-based, being carried out in schools, community centres, health-facilities and even the workplace. This demonstrated targeted education, which is found to be most effective in producing behaviour change compared with universal education [110].

Many of the secondary preventative approaches were carried out in cities or state/province levels. This would be a reflection of the local trauma care system, where a recent review revealed that not many countries have a well-defined, coordinated national trauma system, particularly LMICs, and pre-hospital care would be only available in areas where there are sufficient resources and facilities [105].

Tertiary prevention, although involving mostly rehabilitation centres, also included other settings such as schools, homes and community centres. This is promising, as it enables access to care for neurotrauma patients who live far from such healthcare facilities, which is often the case in LMICs [6].

### Contextual factors

Overall, the contextual factors were discussed in 58 primary studies and 4 reviews, where most of the papers were from HICs with no LMIC papers on tertiary prevention.

Although these factors varied depending on the type of strategy or intervention, there were some commonalities identified. Firstly, the issue of resource. Many papers discussed how the presence or absence of resources determined whether a particular strategy or intervention was utilised or was successful. This was particularly important in LMICs where resources including money and manpower are often constrained or limited, suggesting how approaches which are costly or require many resources may not be successful in these contexts [23, 24]. Conversely, some studies also showed that strategies and interventions that do not require too many resources would be more sustainable and have a wider coverage within the country, contributing to their success.

The second is attitudes and perceptions of individuals towards safety and prevention, and towards the strategy or intervention being implemented. Again, this can be influenced by a number of things including overall awareness or knowledge, age and maturity, peer and family influence or personal experiences. The characteristic of the strategy or intervention also plays a role, especially with tertiary prevention where approaches that were simple, interesting and familiar were more successful. This is particularly important, especially in LMICs, where utilising simple and familiar strategies to rehabilitate, rather than novel, expensive approaches would not only save cost but could potentially lead to good outcomes [91]. Notwithstanding all this, attitudes and perceptions are something that also warrant further exploration.

Another interesting finding from the contextual factors was the relationship between each approach where the presence of one necessitated the other, for example, legislation with enforcement or legislation with awareness concerning legislation (education); and how the absence of one or more resulted in the failure of the other, for example, the absence of policies on vehicle design and engineering resulting in the failure of the utilisation of safety equipment [93].

### Research gaps

This review identified research gaps in neurotrauma and RTCs prevention and the striking difference in the number of publications worldwide. Compared with HICs, there were far less primary studies conducted in the LMICs in all intervention groups, especially tertiary prevention. Moreover, there was a great lack of research from Sub-Saharan Africa, which is the continent with the greatest burden of RTCs [37]. This scenario was also reported in a recent study by Tropeano et al. (2019) which compared publication to traumatic brain injury burden ratio between LMICs and HICs and found it was the lowest in the areas of greatest burden [111]. The underlying reasons may be due to lack of research funding or the absence of relevant strategies and interventions, particularly for rehabilitation, in these countries.

In addition, there were only less than five systematic reviews utilising only LMIC papers. The lack of secondary research to assess and evaluate effectiveness and cost-effectiveness of preventative strategies and interventions is not ideal, where this is important for prioritisation in the midst of resource limitations in these countries [7].

Similarly, not many papers discussed contextual factors that affected or influenced strategy or intervention implementation, especially in LMICs. As contextual factors have been recognised to be crucial for replication or transferability in intervention research, there is a need for more research into this, particularly in LMICs where differences in infrastructure, population, environment and resources can affect the success of a preventative approach [14, 23, 37].

The setting of a strategy and intervention is also essential when looking at transferability and applicability [112]. The fact that many of the review papers did not report the settings of the studied approach makes it difficult to draw conclusions on where it would be effective or useful, and also whether it would be effective in different or multiple settings.

### Strengths and limitations of this scoping review

To our knowledge, this scoping review is the first to identify and quantify the evidence on all types of primary, secondary, and tertiary preventative strategies and interventions for neurotrauma, published in both HICs and LMICs. This review also discusses contextual issues surrounding the implementation of these approaches, which would be useful in making recommendations for policy and practice, particularly in resource-constrained settings.

Additionally, we conducted a comprehensive literature search using five electronic databases which included peer-reviewed literature from a range of disciplines and a wide range of grey literature sources from government and non-governmental websites. Therefore, this review provides a holistic evaluation of preventative strategies and interventions worldwide and a comprehensive comparison and understanding of its relation to the specific settings and/or contexts. Our scoping review is performed with a well-established, rigorous review protocol using a systematic search strategy, thus emphasising reliability and ease of replication.

Our review also included systematic reviews and meta-analyses, which provide information on the types of interventions which have been assessed for effectiveness and which have not. This would influence further research whereby more secondary research can be done for preventative strategies and interventions; and policy, where effective interventions can be implemented as far as possible.

This scoping review is aimed to explore the breadth of evidence in regards to preventative strategies for neurotrauma and RTCs available in both HICs and LMICs. However, LMIC papers were lacking significantly, in spite of grey literature searches, due to the inability to cover websites of all countries. Similarly, we did not perform the grey literature search from all road safety-related databases, but a selected few. The bibliographic and citation searching was limited, and we did not contact researchers for additional papers. We were also aware of the limitations in the number of publications and potential geographical bias in this review due to restriction of the included literature to only English.

As we included a wide range of study types, there was a possibility to include studies with weak methodology. Therefore, findings from this review should be carefully interpreted, particularly in relation to the potential implementation of a preventative strategy or intervention.

Our selection criteria excluded papers that discussed trauma in general which could have potentially limited the number of papers discussing primary and secondary preventative strategies and interventions. Additionally, we did not perform a quality assessment of all the included studies However, despite this limitation, we believe that our robust protocol and screening of literature could safeguard against unreliable information.

The framework used to classify the primary studies on primary prevention was developed using guidance from several existing frameworks [113]. As ours is not an established framework, but one derived from others related to RTCs alone, the accuracy and generalisability of this categorisation for primary neurotrauma prevention may be limited.

## Conclusions

Our scoping review provides the first review about neurotrauma and RTCs preventions evaluated in both HICs and LMICs. Of the 411 included articles, the majority of the studies were performed in HICs. The most common preventative approach was legislation/policy strategies and interventions, followed by helmet use. While HICs and LMICs shared many similar approaches, it appeared that there were several that were absent in LMICs, the most apparent involving technology utilisation. The other obvious deficiency was in pre-hospital care. The settings of the studies were important in understanding the location where the interventions were carried out. The implementation of most primary and secondary prevention was at city, state and national levels while tertiary prevention was mostly carried out at a healthcare or community-based setting. Resource and local attitudes appeared to be the contextual factors influencing the implementation of various strategies and interventions. Our review also emphasises the fact that RTCs are a major cause of neurotrauma globally, and more measures should be put in place to prevent this, particularly in areas of high occurrence. With the deadline for the WHO Sustainable Development Goal (SDG) 3.6 and United Nations’ Decade of Action for Road Safety fast approaching, researchers, especially from LMICs should heed the call to carry out more research into what strategies and interventions in neurotrauma and RTCs prevention exist, and if approaches developed for HICs are applicable to LMICs, and thus be replicated.

## Supplementary information

**Additional file 1.** Preferred reporting items for systematic reviews and meta-analyses extension for scoping reviews (PRISMA-ScR) checklist.

**Additional file 2.** Search strategy for Medline 1946 to date: returned 18,013 records.

**Additional file 3.** List of databases and websites searched for grey literature.

**Additional file 4.** Characteristics of included primary studies after full-text screening (primary and secondary prevention).

**Additional file 5.** Characteristics of included reviews after full-text screening

**Additional file 6.** Definitions/description of rehabilitative strategies and interventions included in the review.

## Data Availability

All data generated and/or analysed during this study are included in this publication [and its Additional files]. Any unpublished material is available from the corresponding author upon request.

## Abbreviations

TBI: Traumatic brain injury
RTCs: Road traffic collisions
LMICs: Low- and middle-income countries
HICs: High-income countries
PCC: Population, Concept, Context
NIHR: National Institute for Health Research
USA: United States of America
UK: United Kingdom
GDLS: Graduated Driver Licensing system
EMS: Emergency Medical Services WHO: World Health Organisation
SDG: Sustainable Development Goal
