# Supplementary material for: Mapping global evidence on strategies and interventions in neurotrauma and road traffic collisions prevention: a scoping review
Source: Syst Rev. 2020 May 20;9:114. doi: 10.1186/s13643-020-01348-z (PMC7240915; doi:10.1186/s13643-020-01348-z)
Supplement: Supplementary file 2 — Additional file 2. Search strategy for Medline 1946 to date: returned 18,013 records. [file 13643_2020_1348_MOESM2_ESM.docx]

**ADDITIONAL FILE 2**

**SEARCH STRATEGY FOR MEDLINE 1946 to date: returned 18013 records**

1. Brain Injuries, Traumatic/ or Craniocerebral Trauma/
2. (neurotrauma or “traumatic brain injur*” or “head injur*” or “acquired brain injur*” or “craniocerebral trauma”).ti,ab.
3. 1 or 2
4. Accidents, Traffic/
5. (“road traffic injur*” or “road traffic collision* or “road traffic accident*” or “road traffic crash*” or “road crash*” or “road accident*” or “road collision*” or “road injur*” or “traffic accident*” or “traffic collision*” or “traffic crash*” or “traffic injur*” or “motor vehicle accident*” or “motor vehicle collision*” or “motor vehicle crash*”). ti,ab.
6. 4 or 5
7. 3 or 6
8. Protective Devices/
9. Primary Prevention/
10. Secondary Prevention/
11. Tertiary Prevention/
12. Environment Design/
13. Law Enforcement/
14. Accident Prevention/or Safety/
15. Emergency Medical Services/
16. Health Services Accessibility/
17. Triage/
18. First Aid/
19. Oxygen/or Oxygen Inhalation Therapy/
20. Rehabilitation/or Neurological Rehabilitation/
21. Mindfulness/
22. (“trauma prevent*” or “accident prevent*” or “crash prevent*” or “injury prevent*” or “prevent* strategy*” or “prevent* intervention*” or “prevent* polic*” or “prevent* programme*” or “prevent* initiative*” or “primary prevent*” or “secondary prevent*” or “tertiary prevent*” or “protective device*” or “environmental design” or “traffic calming system” or “traffic law enforcement” or “law enforcement” or “traffic legislation” or legislation or “graduated driv* licen*” or “access to care” or “health service* accessibility” or “pre-hospital tracheal intubat*” or “pre-hospital transfer” or “pre-hospital triage” or triage or “first aid” or oxygen or “oxygen inhalation* therap*” or “pre-hospital resuscitati*” or “trauma system” or “emergency medical system” or “emergency medical service*” or safety or “road safety” or “traffic safety” or rehab* or “cognitive rehab*” or “psychological rehab*” or “physical rehab*” or “behavio?r* rehab*” or “neurological rehab*” or mindfulness).ti,ab.
23. Or/8-22
24. 7 and 23
25. Limit 24 to (english language and humans and yr=”1974-Current”)
