# Supplementary material for: Mapping global evidence on strategies and interventions in neurotrauma and road traffic collisions prevention: a scoping review
Source: Syst Rev. 2020 May 20;9:114. doi: 10.1186/s13643-020-01348-z (PMC7240915; doi:10.1186/s13643-020-01348-z)
Supplement: Supplementary file 3 — Additional file 3. List of databases and websites searched for grey literature. [file 13643_2020_1348_MOESM3_ESM.docx]

**ADDITIONAL FILE 3**

**LIST OF DATABASES AND WEBSITES SEARCHED FOR GREY LITERATURE**

1. Open Grey ([www.opengrey.eu](http://www.opengrey.eu))
2. The Grey Literature Report ([www.greylit.org/](http://www.greylit.org/))
3. National Institute for Health and Care Excellence (NICE, <https://www.nice.org.uk/>)
4. Global Alliance for NGOs for Road Safety (<http://roadsafetyngos.org/>)
5. World Federation of Neurosurgical Societies (<https://www.wfns.org/>)
6. Fédération Internationale de l’Automobile Foundation (<https://www.fiafoundation.org/>)
7. United Nations Department of Safety and Security (<https://www.un.org/undss/roadtosafety>)
8. European Transport Safety Council (<https://etsc.eu/tag/road-safety/>)
9. Global Road Safety Initiative (<https://www.grsproadsafety.org/resources/>)
10. World Health Organisation (<https://www.who.int/>)
11. Youth for Road Safety (<http://www.youthforroadsafety.org/>)
12. Safe Kids worldwide (<https://www.safekids.org/>)
13. Think First (<https://www.thinkfirst.org/>)
