# Supplementary material for: Mapping global evidence on strategies and interventions in neurotrauma and road traffic collisions prevention: a scoping review
Source: Syst Rev. 2020 May 20;9:114. doi: 10.1186/s13643-020-01348-z (PMC7240915; doi:10.1186/s13643-020-01348-z)
Supplement: Supplementary file 4 — Additional file 4. Characteristics of included primary studies after full-text screening (primary and secondary prevention). [file 13643_2020_1348_MOESM4_ESM.docx]

**ADDITIONAL FILE 4**

**CHARACTERISTICS OF INCLUDED PRIMARY STUDIES AFTER FULL-TEXT SCREENING (Primary and Secondary prevention)**

1. Primary Prevention

| **Intervention/strategy** | **Country type** | **First author, Year, Country** | **Setting** |
| --- | --- | --- | --- |
| **PERSONAL SAFETY/PROTECTIVE EQUIPMENT** | | | |
| Helmets for bicycles and motorised two- or four-wheelers | HIC | Dinh, 2013, Australia | City |
|  |  | Dorsch, 1987, Australia | Bicycling clubs |
|  |  | Finvers, 1996, Canada | Province |
|  |  | Friedman, 2016, Canada | City |
|  |  | Germeni, 2009, Greece | City |
|  |  | Howland, 1989, USA | School |
|  |  | Kim, 2016, Korea | City |
|  |  | Kim, 2018, Korea | City |
|  |  | Krauss, 1996, USA | Counties |
|  |  | Kuo, 2017, Taiwan | City |
|  |  | Luna, 1981, USA | City |
|  |  | Murdock, 1991, USA | State |
|  |  | Ohlin, 2017, Sweden | National |
|  |  | Pierce, 2014, USA | City |
|  |  | Powell, 2004, USA | National |
|  |  | Ranney, 2010, USA | City |
|  |  | Rivara, 1998, USA | National |
|  |  | Rivara, 1999, USA | City |
|  |  | Rodgers, 1990, USA | National |
|  |  | Rutledge, 1993, USA | State |
|  |  | Sethi, 2015, USA | City |
|  |  | Sijits, 1995, The Netherlands | Cities |
|  |  | Singleton, 2017, USA | State |
|  |  | Sullins, 2014, USA | County |
|  |  | Sung, 2016, Korea | Rural area |
|  |  | Thomas, 1994, Australia | City |
|  |  | Thompson, 1996, USA | City |
|  |  | Tsai 1995, Taiwan | City |
|  |  | Van Camp, 1998, Belgium | City |
|  |  | Wasserman, 1990, USA | National |
|  | LMIC | Bhatti, 2010, Pakistan | City |
|  |  | Gupta, 2018, Cambodia | City |
|  |  | Gururaj,2016, India | City |
|  |  | Merali, 2018, Cambodia | Provinces |
|  |  | Phuenpathom, 2001, Thailand | Municipality |
|  |  | Roehler, 2013, Cambodia | City and rural areas |
|  |  | Siviroj, 2012a, Thailand | Provinces |
|  |  | Sreedharan, 2010, India | State |
| Helmets for sports | HIC | Bier, 2018, Germany | Cities |
|  |  | Emery, 2018, Canada | Not reported |
|  |  | Lemione, 2017, USA | State |
|  |  | Le Sage, 2018, Canada | Province |
|  |  | Macnab, 2002, Canada | City |
|  |  | Powell, 2004, USA | National |
|  |  | Short, 2018, USA | City |
|  |  | Sulhei, 2017, Norway | National |
| Occupational helmet use | HIC | Powell, 2004, USA | National |
|  |  | Wallace, 2012, Australia, USA | Not reported |
|  |  | Warren, 2017, Australia | City |
| Seatbelts | HIC | Han, 2017, USA | State |
|  | LMIC | Ng, 2013, Malaysia | National |
|  |  | Sadeghnejad, 2014, Iran | City |
|  |  | Siviroj, 2012b, Thailand | Provinces |
| Child car seat/restraint | HIC | Hunter, 2017, Australia | State |
|  |  | Park, 2018, Korea | National |
|  | LMIC | Karbakhsh, 2016, Iran | City |
|  |  | Soori, 2015, Iran | National |
| Conspicuity equipment | HIC | Lahrmann, 2018, Denmark | National |
|  |  | Tin Tin, 2015, NZ | National |
|  | LMIC | Bacchieri, 2010, Brazil | City |
| Mouth guards, braces, face shields | HIC | Emery, 2018, Canada | Not reported |
| **EDUCATION/TRAINING/AWARENESS-RAISING** | | | |
| Driver or motorcycle rider education/training | HIC | Bidasca, 2015, Europe | National |
|  |  | Ekeh, 2013, USA | Hospital |
|  |  | Gregersen, 1996, Sweden | Workplace |
|  |  | Nasvadi, 2007, Canada | Cities |
|  |  | Ramos, 2008, Spain | City |
|  |  | Salminen, 2008, Finland | Workplace |
|  |  | Shell, 2015, USA | State |
|  |  | Small, 2008, USA | Not reported |
|  |  | West, 2014, USA | Community |
|  |  | Wood, 2010, UK | Not reported |
|  | LMIC | National Highways and Motorways Police, 2017, Pakistan | National |
|  |  | Swaddiwudhipong, 1998, Thailand | District |
| Pedestrian/Bicyclist education | HIC | Myint, 1993, Singapore | Primary schools |
|  |  | Rivara, 1998, USA | National |
|  |  | Schwebel, 2014, USA | City |
|  |  | Utley, 2010. USA | Schools |
|  |  | Violano, 2009, USA | City |
|  | LMIC | Bacchieri, 2010, Brazil | Community centre |
|  |  | Lobosky, 1997, Mexico | High School |
|  |  | Poswayo, 2018, Tanzania | Schools |
| Road safety education (general) | HIC | Yang, 2003, Korea | National |
|  | LMIC | Montero, 2012, Indonesia | City |
|  |  | National Highways and Motorways Police, 2017, Pakistan | National |
|  |  | Salvarani 2009, Brazil | City |
|  |  | Subramaniam, 1989, Malaysia | Not reported |
| Child passenger safety information | HIC | Gielen, 2015, USA | Cities |
|  |  | West, 2014, USA | Communities |
| Education on abusive head trauma in infants | HIC | Dias, 2005, USA | Hospital |
|  |  | Jenny, 2009, USA | Not reported |
|  |  | Samaha, 2015, USA | Healthcare centre, Home |
|  |  | Stewart, 2015, Canada | Province |
|  |  | Zolotor, 2015, USA | State |
| Sports safety training/education | HIC | Swartz, 2015, USA | State |
|  | LMIC | Brown, 2016, South Africa | City |
| Road safety Campaigns | HIC | Guttman, 2016, Israel | Not reported |
|  |  | Iancu, 1993, Israel | National |
|  |  | Jagim, 1997, USA | National |
|  |  | Layba, 2017, USA | High school |
|  |  | Miller, 2004, NZ | National |
|  |  | Samaha, 2015, USA | National |
|  |  | Stevenson, 2014, Australia | National |
|  |  | Stojanova, 2018, Czech Republic | National |
|  |  | Wesson, 2000, Canada | Schools and community |
|  |  | West, 2014, USA | Communities |
|  |  | Zampetti, 2013, Italy | Province |
|  | LMIC | Cawich, 2010, Jamaica | National |
|  |  | Cohen, 2008, Colombia | City |
|  |  | National Highways and Motorways Police, 2017, Pakistan | National |
|  |  | Salvarani, 2009, Brazil | City |
|  |  | Slesak, 2011, Laos | Province |
|  |  | Soori, 2009, Iran | National |
| Abusive Head Trauma Campaigns | HIC | Stewart, 2011, Canada | Province |
|  |  | Stewart, 2015, Canada | Province |
|  |  | Zolotor, 2015, USA | State |
| **LEGISLATION/POLICY** | | | |
| Helmet laws/policies (motorised vehicles and bicyclists) | HIC | Bonander, 2014, Sweden | National |
|  |  | Dennis, 2013, Canada | Provinces and territories |
|  |  | Fleming, 1992, USA | State |
|  |  | Hassan, 2014, USA | States |
|  |  | Jagim, 1997, USA | National |
|  |  | Ji, 2006, USA | County |
|  |  | Krauss, 1996, USA | Counties |
|  |  | Lee, 2005, USA | State |
|  |  | Macpherson, 2002, Canada | Provinces |
|  |  | Mann, 1999, UK | Town |
|  |  | Markowitz, 2015, USA | States and districts |
|  |  | McDermott, 1995, Australia | State |
|  |  | Naumann, 2015, USA | States |
|  |  | Nurchi, 1987, Italy | Province |
|  |  | Olsen, 2015, USA | States |
|  |  | Pardi, 2007, USA | State |
|  |  | Scuffham, 2000, NZ | National |
|  |  | Shafi, 1998, USA | State |
|  |  | Walter, 2011, Australia | State |
|  |  | Williams, 2018, USA | State |
|  | LMIC | Abegaz, 2014, Ethiopia | State |
|  |  | Craft, 2017, Vietnam, Cambodia and Uganda | Communities |
|  |  | Ha, 2018, Vietnam | District |
|  |  | Ichikawa, 2003, Thailand | Province |
|  |  | Khan, 2014, Pakistan | National |
|  |  | Olson, 2016, Vietnam | National |
|  |  | Passmore, 2010, Vietnam | Provinces |
|  |  | Ralaidovy, 2018, Southeast Asia and Sub-saharan Africa | National |
|  |  | Venturini, 2019, Cambodia | City |
|  |  | WHO, 2015, Russia | National |
| Graduated driver licensing system | HIC | Begg, 2001, NZ | Region |
|  |  | Chen, 2006, USA | States |
|  |  | Cheng, 2012, USA | State |
|  |  | Conner, 2016, USA | State |
|  |  | Ehsani, 2013, USA | States |
|  |  | Foss, 2001, USA | State |
|  |  | Hallmark, 2008, USA | State |
|  |  | Hyde, 2005, USA | State |
|  |  | Jagim, 1997, USA | National |
|  |  | Langford, 2004, Australia | Cities |
|  |  | Langley, 1996, NZ | National |
|  |  | Masten, 2010, USA | State |
|  |  | McCartt, 2001, USA | State |
|  |  | McCartt, 2010, USA | States |
|  |  | Neyens, 2008, USA | State |
|  |  | O’Connor, 2016, USA | State |
|  |  | Reeder, 1999, NZ | National |
|  |  | Rogers, 2011, USA | States |
|  |  | Senserrick, 2018, Australia | State |
|  |  | Shope, 2001, USA | State |
|  |  | Shope, 2004, USA | State |
|  |  | Taubman, 2011, Israel | National |
|  |  | Toledo, 2011, Israel | National |
|  |  | Ulmer, 2000, USA | State |
|  |  | Williams, 2017, USA | National |
|  | LMIC | Cawich, 2010, Jamaica | National |
|  |  | WHO, 2015, Russia | National |
| Seatbelt and child passenger safety laws/policies | HIC | Agran, 1987, USA | County |
|  |  | Kuo, 2015, Taiwan | National |
|  |  | Jagim, 1997, USA | National |
|  |  | Petty, 1975, Australia | City |
|  |  | Stevenson, 2014, Australia | National |
|  |  | Thomas, 1990, UK | National |
|  | LMIC | Abegaz, 2014, Ethiopia | State |
|  |  | Khan, 2014, Pakistan | National |
|  |  | Ralaidovy, 2018, Southeast Asia and Sub-saharan Africa | National |
|  |  | Soori, 2011, Iran | National |
|  |  | WHO, 2015, Russia | National |
| Policies/legislation on drink-driving | HIC | Brubacher, 2017, Canada | State |
|  |  | Jagim, 1997, USA | National |
|  |  | Miller, 2004, NZ | National |
|  |  | Nagata, 2006, Japan | Prefectures |
|  |  | Stevenson,2014, Australia | National |
|  | LMIC | Abegaz, 2014, Ethiopia | State |
|  |  | Chandran, 2014, Mexico | Cities |
|  |  | Ralaidovy, 2018, Southeast Asia and Sub-saharan Africa | National |
|  |  | Sebego, 2014, Botswana | National |
|  |  | WHO, 2015,Russia | National |
| Policies/legislation on speed or speeding | HIC | Engel, 1992, Denmark | Residential areas |
|  |  | Grundy, 2009, UK | City |
|  |  | Ohlin, 2017, Sweden | National |
|  |  | Ramos, 2008, Spain | City |
|  |  | ROSPA, 2019, UK | Cities |
|  |  | Vadeby, 2018, Sweden | National |
|  | LMIC | Abegaz, 2014, Ethiopia | State |
|  |  | He, 2018, China | Province |
|  |  | Poli de Figueiredo, 2001, Brazil | National |
|  |  | Ralaidovy, 2018, Southeast Asia and Sub-saharan Africa | National |
|  |  | WHO, 2015, Russia | National |
| Cell-phone/texting bans | HIC | Ferdinand, 2015, USA | States |
|  |  | Kwon, 2014, USA | State |
|  | LMIC | Abegaz, 2014, Ethiopia | State |
| Policies on fitness to drive and licensing restrictions | HIC | Bidasca, 2015, Europe | Workplace |
|  |  | Iancu, 1993,Israel | Not reported |
|  |  | Langford, 2011, Australia | State |
|  |  | Small, 2008, USA | Not reported |
| Policies on safe driving in the workplace | HIC | Bidasca, 2015, Europe | Workplace |
|  | LMIC | Abegaz, 2014, Ethiopia | State |
| Policies on vehicle and road user conspicuity | HIC | Hollo, 1998, Hungary | National |
|  |  | Yuan, 2000, Singapore | National |
| Road Safety laws (general) | HIC | Jagim, 1997, USA | National |
|  |  | Lieutaud, 2016, France | Region |
|  |  | Nistal-Nuno, 2017, Chile | National |
|  |  | Rivara, 1998, USA | National |
|  | LMIC | Cawich, 2010, Jamaica | National |
|  |  | Poli de Figueiredo, 2001, Brazil | National |
|  |  | Sebego, 2014, Botswana | National |
|  |  | Sheng, 2018, China | City |
| Road safety audit | HIC | Moses, 1992, USA | National |
| Policies for road and vehicle engineering | HIC | Moran, 2017, Germany | National |
|  |  | Yannis, 2016, Europe | Cities and rural areas |
| Traffic signs/symbols | HIC | Saric, 2018, Croatia | National |
|  |  | Shinar, 2003, Canada, Israel, Finland, Poland | National |
| Crossing guards | HIC | Rothman, 2015, Canada | City |
| Congestion charging scheme | HIC | Li, 2012, UK | City |
| Rewards for good driving or reporting unsafe driving | HIC | Gregersen, 1996, Sweden | Workplace |
|  |  | Yang, 2003, Korea | National |
| **ENFORCEMENT** | | | |
| Traffic policing/patrolling by police | HIC | Beenstock, 2001, Israel | National |
|  |  | Davis, 2006, USA | County |
|  |  | Wood, 2010, UK | Not reported |
|  |  | Yannis, 2007, Greece | National |
|  | LMIC | Bishai, 2014, Uganda | City |
|  |  | Cohem, 2008, Colombia | City |
|  |  | Khan, 2014, Pakistan | National |
|  |  | Soori, 2009, Iran | National |
|  |  | Tetali, 2013, India | City |
|  |  | WHO, 2015, Russia | National |
| Photo enforcement including enforcement of speed limits | HIC | Blais, 2015, France | National |
|  |  | Goldenbeld, 2005, The Netherlands | Province |
|  |  | Hoye, 2015, Norway | National |
|  |  | Jeffrey, 2010, UK | City |
|  |  | Ko, 2017, USA, | City |
|  |  | Llau, 2015, USA | County |
|  |  | Montella, 2015, Italy | City |
|  |  | Morain, 2016, USA | States |
|  |  | Mountain, 2005, USA | National |
|  |  | NCSL, 2018, USA | States |
|  |  | Novoa, 2010, Spain | City |
|  |  | Shin, 2007, USA | City |
|  |  | Stevenson, 2014, Australia | National |
|  |  | Testerman, 2013, USA | National |
|  |  | Vanlaar, 2014, Canada | City |
|  |  | Yang, 2003, Korea | National |
|  | LMIC | Ralaidovy, 2018, Southeast Asia and Sub-saharan Africa | National |
|  |  | WHO, 2015, Russia | National |
| Enforcement of drink and drug driving | HIC | Levy, 1989, USA | State |
|  |  | Miller, 2004, NZ | National |
|  |  | Stevenson, 2014, Australia | National |
|  |  | Tay, 2005, Australia | State |
|  |  | West, 2014, USA | Communities |
|  | LMIC | Chandran, 2014, Mexico | Cities |
|  |  | Ralaidovy, 2018, Southeast Asia and Sub-saharan Africa | National |
| Penalties, fines and demerit point system for traffic offenders; criminalization of traffic offenses | HIC | Novoa, 2011, Spain | National |
|  |  | Stevenson, 2014, Australia | National |
|  |  | Yang, 2003, Korea | National |
|  | LMIC | Cawich, 2010, Jamaica | National |
|  |  | WHO, 2015, Russia | National |
|  |  | West, 2014, USA | Communities |
|  |  | Ralaidovy, 2018, Southeast Asia and Sub-saharan Africa | National |
|  |  | Soori, 2009, Iran | National |
| Enforcement of laws on use of helmets, seat belts and child car safety seats | HIC | West, 2014, USA | Communities |
|  | LMIC | Ralaidovy, 2018, Southeast Asia and Sub-saharan Africa | National |
|  |  | Soori, 2009, Iran | National |
| **ENGINEERING** | | | |
| Traffic calming and road modification or redesign measures | HIC | Choi, 2018, Korea | City |
|  |  | Dimaggio, 2013, USA | City |
|  |  | Faure, 1992, France | Cities |
|  |  | Gross, 2013, USA | States |
|  |  | Herrstedt, 1992, Denmark | Towns |
|  |  | Johansson, 2009, Sweden | National |
|  |  | Jones, 2012, UK | Wards |
|  |  | Leden, 2006, Sweden | Cities |
|  |  | Lord, 2007, USA | States |
|  |  | Mountain, 2005, UK | National |
|  |  | NICE, 2010, UK | National |
|  |  | Vieira, 2012, Portugal | City |
|  |  | Wood, 2010, UK | Not reported |
|  |  | Yannis, 2014, Portugal | Municipality |
|  | LMIC | Cohen, 2008, Colombia | City |
|  |  | Khan, 2014, Pakistan | National |
|  |  | Nadesan-Redy, 2013, South Africa | Residential areas |
|  |  | Poswayo, 2018, Tanzania | City |
|  |  | Subramaniam, 1989, Malaysia | Not reported |
|  |  | Tetali, 2013, India | City |
|  |  | WHO, 2015, Russia | National |
| Traffic signals and pedestrian countdown signals | HIC | Atta Boateng, 2018, USA | State |
|  |  | Escott, 2017, Canada | City |
|  |  | Persaud, 1997, USA | City |
|  |  | Song, 2019, USA | City |
|  |  | Szeto, 1991, USA | Cities |
|  | LMIC | WHO, 2015, Russia | National |
| Road lighting | HIC | Wanvik, 2009, The Netherlands | National |
| Pedestrian footbridge | LMIC | Hasan, 2018, Malaysia | City |
| Audio-tactile lane marking | HIC | Hatfield, 2009, Australia | State |
| Exclusive lanes | HIC | Dimaggio, 2013, USA | City |
|  |  | Morrison, 2019, Australia | City |
|  |  | Pulugurtha, 2015, USA | City |
|  |  | Wood, 2010, UK | Not reported |
|  | LMIC | Cohen, 2008, Colombia | City |
|  |  | Osorio-Cuellar, 2017, Colombia | City |
| Vehicle design | HIC | Ohlin, 2017, Sweden | National |
|  |  | Page, 2011, France | National |
| In-vehicle safety technology | HIC | Jagger, 1987, USA | State |
|  |  | Kullgren, 2005, Europe | Not reported |
|  |  | Pintar, 2000, USA | National |
|  |  | Stuke, 2010, USA | Not reported |
| Crash avoidance technology/measures | HIC | Bidasca, 2015, Europe | Workplace |
|  |  | Broughton, 2002, UK | Not reported |
|  |  | Cicchino, 2018, USA | States |
|  |  | Evans, 1996, USA | States |
|  |  | Isaksson-hellman, 2016, Sweden | National |
|  |  | Kullgren, 2005, Europe | Not reported |
|  |  | Ohlin, 2017, Sweden | National |
|  |  | Page, 2011, France | National |
|  |  | Rizzi, 2015, Sweden, Italy, Spain | National |
|  |  | Rizzi, 2016, Sweden | National |
|  |  | Sternlund, 2017, Sweden | National |
|  |  | Stevenson, 2014, Australia | National |
|  | LMIC | Bacchieri, 2010, Brazil | Community centre |
|  |  | Cawich, 2010, Jamaica | National |
|  |  | Khorasani-Zavareh, 2013, Iran | National |
| **MULTI-COMPONENT** | | | |
| Harlem Hospital Injury Prevention Programme (engineering, education, policies) | HIC | Durkin, 1999, USA | Neighbourhoods |
| Motor Vehicle Injury Prevention Programme (education, enforcement, policy/legislation) |  | Pontkowski, 2015, USA | Communities |
| The Harstad Injury Prevention Programme (education, legislation/policy, engineering, enforcement) |  | Ytterstad, 1994, Norway | Cities |
| Motorcycle safety programme (safety equipment, education, engineering, policies/legislation) | LMIC | Radin, 2006, Malaysia | National |

1. Secondary prevention

| **Intervention/strategy** | **Country type** | **First author, Year, Country** | **Setting** |
| --- | --- | --- | --- |
| Prehospital airway management | HIC | Bernard, 2010, Australia | Cities |
|  |  | Bukur, 2011, USA | County |
|  |  | Cooper, 2011, USA | National |
|  |  | Davis, 2005, USA | County |
|  |  | Denninghoff, 2017, USA | National |
|  |  | Karamanos, 2014, USA | Counties |
|  |  | Lansom, 2016, Australia | City |
|  |  | Murray, 2000, USA | County |
|  |  | Silverston, 1989, UK | Province |
|  |  | Topping, 2006, USA | State |
|  |  | Warner, 2007, USA | City |
| Pre-hospital fluid resuscitation | HIC | Hernandez, 2017, USA | City |
| Pre-hospital triage/imaging | HIC | Bouzat, 2015, France | Regional area |
|  |  | Schulman, 2007, USA | City |
|  |  | Schwindling, 2016, UK | County |
| Ambulance services | HIC | Al-Shaqsi, 2014, Oman | National |
|  |  | Jones, 1995, UK | County |
|  |  | Lin, 2017, Singapore | National |
|  | LMIC | Fan, 2018, India | National |
|  |  | Jamshidi, 2019, Iran | Not reported |
|  |  | Mahama, 2018, Ghana | Region |
|  |  | Subramaniam, 1989, Malaysia | Not reported |
|  |  | Vasudevan, 2016, India | States |
| Air EMS | HIC | Bekelis, 2015, USA | Hospitals |
|  |  | Cameron, 1993, Australia | State |
|  |  | De Jongh, 2012, The Netherlands | County |
|  |  | Elswick, 2018, USA | Not reported |
|  |  | Landreau, 2018, Argentina | City |
|  |  | Pakkanen, 2017, Finland | Districts |
|  |  | Park, 2014, Korea | City |
|  |  | Sun, 2017, USA | National |
|  |  | Tsai, 2006, Taiwan | Remote islands |
|  | LMIC | Salimi, 2009, Iran | City |
| Physician staffed/physician-led EMS | HIC | Franschman, 2012, The Netherlands | Cities |
|  |  | Hesselfedlt, 2013, Denmark | Cities |
|  |  | Pakkanen, 2016, Finland | Districts |
|  |  | Pakkanen, 2019, Finland | Districts |
|  |  | REVIVE, 2019, Europe | National |
|  |  | Silverston, 1985, UK | Province |
|  | LMIC | Macmahon, 1974, South Africa | City |
| Pre-hospital care training | HIC | Blomberg, 2013, Sweden | Hospitals |
|  |  | Jagim, 1987, USA | National |
|  |  | Langram, 2006, UK | State |
|  | LMIC | Delaney, 2018, Uganda | School |
|  |  | Vakili, 2014, Iran | Province |
|  |  | Vyas, 2016, India | Educational institutions |
|  |  | WHO, 2015, Russia | National |
| Direct transport to neurosurgical centre/theatre | HIC | Lecky, 2016, UK | Counties |
| Organised trauma/emergency medical system | HIC | Gabbe, 2011, Australia, UK | State |
|  |  | Tallon, 2012, Canada | Province |
|  | LMIC | Murad, 2012, Iraq | Province |
|  |  | Riyapan, 2018, Thailand | City |
| Lay first responders | HIC | Sinclair, 1991, USA | National |
|  | LMIC | Arellano, 2010, Dominican Republic | Province |
| Crash notification and response systems | HIC | Gonzalez, 2009, USA | State |
|  |  | Spivak, 1998, USA | City |
|  |  | Stickles, 2018, USA | National |
| Multiple interventions | HIC | Davies, 2006, UK | Not reported |
|  |  | Nelson, 2015, USA | State |
|  |  | Tobin, 2017, USA, UK | Not reported |
