# Supplementary material for: Mapping global evidence on strategies and interventions in neurotrauma and road traffic collisions prevention: a scoping review
Source: Syst Rev. 2020 May 20;9:114. doi: 10.1186/s13643-020-01348-z (PMC7240915; doi:10.1186/s13643-020-01348-z)
Supplement: Supplementary file 5 — Additional file 5. Characteristics of included reviews after full-text screening [file 13643_2020_1348_MOESM5_ESM.docx]

**ADDITIONAL FILE 5**

**CHARACTERISTICS OF INCLUDED REVIEWS AFTER FULL-TEXT SCREENING**

1. Primary prevention

**Single Interventions**

| **Intervention/strategy** | **Country type** | **First author, Year** | **Setting** |
| --- | --- | --- | --- |
| **PERSONAL SAFETY/PROTECTIVE EQUIPMENT** | | | |
| Helmets for bicycles and motorised two-or four-wheelers | LMIC and HIC | Liu, 2008 | Not Reported |
|  |  | Olivier, 2017 | Not reported |
|  |  | Thompson, 1999 | Cities |
| Helmets for sports | HIC | Kwiatkowski, 2015 | Not Reported |
|  |  | McIntosh, 2011 | Countries |
| **EDUCATION/TRAINING/AWARENESS-RAISING** | | | |
| High school driver education | HIC | Vernick, 1999 | Cities |
| Road safety mass media campaign | HIC | Hutchinson, 2011 | Country |
|  |  | Phillips, 2011 | City, State, Workplace, National |
| Motorcycle rider training | LMIC and HIC | Kardamanidis, 2010 | Not reported |
| Mindfulness | LMIC and HIC | Koppel, 2019 | Not reported |
| **LEGISLATION/POLICY** | | | |
| Graduated driver licensing | HIC | Foss, 1999 | Not reported |
|  |  | Hartling, 2004 | Not reported |
|  |  | Russell, 2011 | Cities |
|  |  | Zhu, 2013 | States, Counties |
| Bicycle helmet legislation | HIC | Hoye, 2018 | Not reported |
|  |  | Macpherson, 2008 | Cities, Counties |
| Driver licensing restriction | HIC | O Bryne, 2015 | Not reported |
| **ENFORCEMENT** | | | |
| Speed cameras | HIC | Pilkington, 2005 | Not reported |
|  |  | Soole, 2013 | Cities |
|  |  | Willis, 2010 | National |
| Red light cameras | HIC | Aeron-Thomas, 2005 | Country, City, State |
|  |  | Erke, 2009 | Cities |
|  |  | Llau, 2014 | Cities |
|  |  | Retting, 2003 | Cities |
| **ENGINEERING** | | | |
| Street lighting | HIC | Beyer, 2009 | Streets |
| Bus Rapid Transit | LMIC and HIC | Vecino-Ortiz, 2015 | Cities |

**Multiple Interventions**

| **Intervention/strategy** | **Country type** | **First author, Year** | **Setting** |
| --- | --- | --- | --- |
| **PERSONAL SAFETY/PROTECTIVE EQUIPMENT** | | | |
| Helmets for bicycles and motorised two-or-four wheelers | LMIC | Bonnet, 2018 | School-based, Community, Healthcare centres |
|  |  | Forjouh, 2003 | Not reported |
|  | LMIC and HIC | Araujo, 2017 | Multiple |
| Helmets for sports | HIC | Benson, 2011 | School-based, Ski resorts, National Sports Council |
| Mouth-guards, face shield | HIC | Benson, 2011 | School-based, Ski resorts, National Sports Council |
| Child car seat | HIC | Ishikawa, 2014 | Community |
| Seatbelt | LMIC | Forjouh, 2003 | Not reported |
| Reflective clothing | LMIC and HIC | Araujo, 2017 | Multiple |
| **EDUCATION/TRAINING/AWARENESS-RAISING** | | | |
| Compulsory training before and after license | LMIC and HIC | Araujo, 2017 | Multiple |
| Education on road safety (general) | LMIC | Bonnet, 2018 | School-based, Community, Healthcare centres |
|  |  | Staton, 2016 | Not reported |
|  | LMIC and HIC | Ghaffar, 2002 | Not reported |
|  |  | Lefio, 2018 | Not reported |
|  | HIC | Short, 2014 | School-based, Community |
| Education on child seat | HIC | Ishikawa, 2014 | Community |
| Education on abusive head trauma | HIC | Lopes, 2018 | Not reported |
| Early Parenting Program | HIC | Lopes, 2018 | Not reported |
| Driver education | LMIC | Forjouh, 2003 | Not reported |
| **LEGISLATION/POLICY** | | | |
| Helmet laws/policies for bicyclists and motorised vehicle users | LMIC | Staton, 2016 | Not reported |
|  | LMIC and HIC | Araujo, 2017 | Multiple |
|  |  | Hijar, 2012 | Countries |
|  | HIC | Rattan, 2018 | Cities |
| Policies/legislation on drink-driving | LMIC | Forjouh, 2003 | Not reported |
|  | LMIC and HIC | Araujo, 2017 | Multiple |
|  |  | Hijar, 2012 | Countries |
|  |  | Lefio, 2018 | Not reported |
| Licensing restrictions | LMIC and HIC | Ghaffar, 2002 | Not reported |
| Graduated Driver Licensing | LMIC | Forjouh, 2003 | Not reported |
|  | LMIC and HIC | Lefio, 2018 | Not reported |
|  |  | Porchia, 2014 | Countries, Cities |
| Licensure suspension law | LMIC | Forjouh, 2003 | Not reported |
| Sobriety law, blood alcohol content reduction law | LMIC | Staton, 2016 | Not reported |
|  |  | Forjouh, 2003 | Not reported |
|  | LMIC and HIC | Ghaffar, 2002 | Not reported |
| Seatbelt and child passenger safety laws/policies | LMIC | Forjouh, 2003 | Not reported |
|  |  | Staton, 2016 | Not reported |
|  | LMIC and HIC | Ghaffar, 2002 | Not reported |
|  |  | Hijar, 2012 | Countries |
|  |  | Lefio, 2018 | Not reported |
| Policies/legislation on speed or speeding | LMIC and HIC | Hijar, 2012 | Countries |
|  |  | Lefio, 2018 | Not reported |
| Road safety law (general) | LMIC | Bonnet, 2018 | School-based, Community, Healthcare centres |
|  |  | Staton, 2016 | Not reported |
|  | LMIC and HIC | Lefio, 2018 | Not reported |
|  | HIC | Ishikawa, 2014 | Community |
|  |  | Rattan, 2018 (Rishi, 2018) | Cities |
| Cell-phone/texting bans | LMIC | Staton, 2016 | Not reported |
| Rewards for good driving | HIC | Short, 2014 | School-based, Community |
| **ENFORCEMENT** | | | |
| Enforcement of speed limits | LMIC | Forjouh, 2003 | Not reported |
|  | LMIC and HIC | Ghaffar, 2002 | Not reported |
| Enforcement of laws on use of helmets, seat belts and child car safety seats | LMIC and HIC | Ghaffar, 2002 | Not reported |
|  | HIC | Short, 2014 | School-based, Community |
| Enforcement of alcohol use check | LMIC | Bonnet, 2018 | School-based, Community, Healthcare centres |
|  |  | Forjouh, 2003 | Not reported |
|  | LMIC and HIC | Lefio, 2018 | Not reported |
| Traffic policing/patrolling | LMIC | Staton, 2016 | Not reported |
|  | HIC | Short, 2014 | School-based, Community |
| Penalties and fines for traffic offenders | LMIC | Staton, 2016 | Not reported |
|  | LMIC and HIC | Araujo, 2017 | Multiple |
| Photo enforcement (e.g. red light cameras) | LMIC | Forjouh, 2003 | Not reported |
| **ENGINEERING** | | | |
| Traffic calming and road modification | LMIC | Bonnet, 2018 | School-based, Community, Healthcare centres |
|  |  | Forjouh, 2003 | Not reported |
|  |  | Staton, 2016 | Not reported |
|  | LMIC and HIC | Araujo, 2017 | Multiple |
|  |  | Ghaffar, 2002 | Not reported |
|  | HIC | Bunn, 2003 | Multiple |
|  |  | Elvik, 2001 | Roads in residential areas and main roads |
| Pedestrian overpass | LMIC | Bonnet, 2018 | School-based, Community, Healthcare centres |
| Exclusive lanes | LMIC | Forjouh, 2003 | Not reported |
| Street lighting | LMIC | Forjouh, 2003 | Not reported |
|  | LMIC and HIC | Lefio, 2018 | Not reported |
|  |  | Porchia, 2014 | Countries, Cities |
| Vehicle engineering | LMIC | Forjouh, 2003 | Not reported |
|  | LMIC and HIC | Araujo, 2017 | Multiple |
|  |  | Ghaffar, 2002 | Not reported |
|  |  | Lefio, 2018 | Not reported |
| Vehicle and road user conspicuity | LMIC | Bonnet, 2018 | School-based, Community, Roads, Healthcare centres |
|  |  | Forjouh, 2003 | Not reported |
|  | LMIC and HIC | Ghaffar, 2002 | Not reported |
|  |  | Porchia, 2014 | Countries, Cities |

1. Secondary Prevention

| **Intervention/strategy** | **Country type** | **First author, Year** | **Setting** |
| --- | --- | --- | --- |
| Trauma system | LMIC | Adeloye, 2012 | Not reported |
| Direct transport to neurosurgical centre | HIC | Pickering, 2015 | Cities |
| Pre-hospital tracheal intubation | HIC | Von Elm, 2009 | Cities |

1. Tertiary Prevention

| **Intervention/strategy** | **Country type** | **First author, Year** | **Setting** |
| --- | --- | --- | --- |
| Telerehabilitation | HIC | Betts, 2018 | Home |
| Technology-based rehabilitations (e.g. computer games, virtual reality, Wii balance board, 3D games, video games, optokinetic simulation postural instability exercise, computerised-cognitive trainings) | LMIC and HIC | Fetta, 2017 | Community, Healthcare centres, Rehabilitation centres |
|  |  | Lindsay, 2015 | Home, Healthcare centres, Rehabilitation centres |
|  |  | Sigmundsdottir, 2016 | Rehabilitation centres |
|  | HIC | Linden, 2016 | Not reported |
|  |  | Maggio, 2019 | Not reported |
|  |  | Pei, 2016 | Not reported |
|  |  | Saywell, 2017 | Rehabilitation centres |
|  |  | Thomas, 2017 | Not reported |
| Cognitive training (e.g. art-based social skills, problem solving, reasoning training, self-management training, daily planning, routine management) | LMIC and HIC | Lindsay, 2015 | Home, Healthcare centres, Rehabilitation centres |
|  | HIC | Clasby, 2018 | Home, Healthcare centres, Cinema, Library, Community |
|  |  | Thomas, 2017 | Not reported |
| Psychological interventions (e.g. Cognitive Behavioural Therapy, psychotherapy, mindfulness) | LMIC and HIC | Lindsay, 2015 | Home, Healthcare centres, Rehabilitation centres |
|  | HIC | Gertler, 2015 | Not reported |
|  |  | Kenuk, 2017 | Not reported |
|  |  | Liu, 2018 | Rehabilitation centres |
|  |  | Stalder-Luthy, 2013 | Not reported |
|  |  | Thomas, 2017 | Not reported |
| Physical interventions (e.g. supervised exercise programme, cycling) | HIC | Gertler, 2015 | Not reported |
|  |  | Liu, 2018 | Rehabilitation centres |
|  |  | Morris, 2016 | Not reported |
|  |  | Pei, 2016 | Not reported |
|  |  | Thomas, 2017 | Not reported |
| Acupuncture | LMIC | Wong, 2011 | Not reported |
| Transcranial magnetic stimulation | HIC | Gertler, 2015 | Not reported |
| Music therapy | LMIC and HIC | Magee, 2017 | Not reported |
| Education about neurotrauma and symptom management | LMIC and HIC | Lindsay, 2015 | Home, Healthcare centres |
|  | HIC | Thomas, 2017 | Not reported |
| Family-supported treatment | LMIC and HIC | Lindsay, 2015 | Home, Healthcare centres, Rehabilitation centres |
| Multidisciplinary rehabilitation | HIC | Pei, 2016 | Not reported |
| Animal assisted therapy | LMIC and HIC | Stapleton, 2016 | Not reported |
