# Supplementary material for: Mapping global evidence on strategies and interventions in neurotrauma and road traffic collisions prevention: a scoping review
Source: Syst Rev. 2020 May 20;9:114. doi: 10.1186/s13643-020-01348-z (PMC7240915; doi:10.1186/s13643-020-01348-z)
Supplement: Supplementary file 6 — Additional file 6. Definitions/description of rehabilitative strategies and interventions included in the review. [file 13643_2020_1348_MOESM6_ESM.docx]

**ADDITIONAL FILE 6**

**DEFINITIONS/DESCRIPTIONS OF REHABILITATIVE INTERVENTIONS AND STRATEGIES INCLUDED IN THE REVIEW**

*Acupuncture* (1)

The best known complementary and alternative medicine that originated in China. Involves the stimulation of specific points on the skin, known as acupoints or meridian points, by the insertion of fine needles. In rehabilitation, it is believed to stimulate small-diameter nerves which lead to multiple circulatory and biochemical effects that would affect various physiological systems in the brain and periphery.

*Animal assisted therapy* (2)

A goal-directed therapy that uses animals as a motivating force to enhance a particular treatment (i.e. speech therapy, occupational therapy) session for the benefit of the client*.*

*Art therapy* (3,4)

This therapeutic technique is rooted in the idea that creative expression helps enhance perceived control, build a sense of self, purpose, and social support and support emotions. It is also believed to promote brain plasticity. It is not limited to drawings or painting but can take the form of any creative expression such as floral arrangement, arts and crafts.

*Cognitive Behavioural Therapy* (5, 6)

Developed in the 1960s by Aaron Beck and sometimes known as cognitive therapy. It aims to correct distortions in cognitive thinking which are attributable to the patient’s perception of an event rather than the event itself. Usually involves talking and can be carried out individually or as a group.

*Cognitive Pragmatic Treatment* (7)

A rehabilitative program that seeks to improve communication competence by training different abilities such as conversational skills, social appropriateness, self-awareness and inferential abilities.

*Cognitive rehabilitation/Traditional cognitive rehabilitation/Cognitive training* (8,9,10)

Structured activities that improve a brain-injured patient’s higher cerebral functioning or help the individual better understand the nature of those difficulties while teaching him or her methods of compensation. These activities would target different areas of cognition such as executive functions, verbal memory, visual memory, spatial memory, visual and spatial abilities, visual attention and processing speed. Different methods can be used for the activities including pen and paper, board games or puzzles, musical instruments and electronic systems.

*Computerised cognitive rehabilitation/computer-assisted cognitive rehabilitation/Technology-based rehabilitation* (11, 12,13)

The use of computers for inducing enduring cognitive change. This may include computer or video games, special rehabilitation software, or simulation (tasks and environment).

*Computer assisted rehabilitation environment (CAREN)* (14)

A multi-sensory virtual reality system that provides simulations to improve physical and cognitive functioning. First developed in the Netherlands, it is used for treatment and rehabilitation of neuropsychological conditions.

*Coordinated multidisciplinary rehabilitation/Multi-disciplinary rehabilitation* (15, 16)

Rehabilitation led by a clinical coordinator and carried out by various healthcare professionals such as physical and occupational therapists, speech therapists, psychologists, social workers, nurses and doctors, with regular team meetings and discussions.

*Exercise therapy* (17, 18)

A regimen or plan of physical activities designed and prescribed for specific therapeutic goals. In neurotrauma, it is believed to cause upregulation of neurotrophins and neurogenesis within the hippocampus.

*Family –supported treatment* (19)

An intervention for children aged 5-12 years where parents observe and take responsibility for their child’s rehabilitation routines. Parents would also attend information sessions and support groups, in addition to daily one-on-one sessions.

*Goal Management Training* (20)

A brief intervention to improve problem solving and planning skills

*Home-based circuitry training* (21)

A set of endurance or resistance training exercises which are completed in a set period of time at home.

*Implementation intentions* (20)

A training technique that encourages brain injured patients to use imagery.

*INSIGHT: Improving Natural Social interaction: Group rehabilitation after Traumatic Brain Injury* (22)

Draws on behaviour management techniques and the development of positive routines and identity reconstruction. This is a client-centred approach that provides a contextualised, flexible and natural environment for group interactions.

*Integrated cognitive rehabilitation* (23)

Cognitive rehabilitation which includes a psychological component of therapy, such as psychotherapy or mindfulness.

*Intelligent Assistant Therapy* (24)

An automatic therapy planning functionality or algorithm which automatically selects, configures and schedules rehabilitation tasks for patients with cognitive impairments after acquired brain injury.

*Interactive Metronome Therapy* (25)

A behavioural feedback operant conditioning system in which a patient executes various repeated movements in time to a beat, while a computer provides precision feedback on performance.

*Mindfulness/Mindfulness-based therapies* (23, 26, 27)

Mindfulness is defined as paying attention, on purpose, to the present moment without judgment. Such therapies teach patients to decentre from problematic thoughts by viewing them as mental events rather than accurate reflections of reality. Often used in combination with other modalities such as cognitive behavioural therapy and yoga.

*Music therapy/Music interventions/Music training/Neurologic music therapy/music-based cognitive remediation therapy* (28, 29, 30, 31)

The therapeutic application of music to restore, maintain and/or improve physical, emotional, psychosocial and neurological function. It ranges from passive techniques such as listening to songs to active music making, composition and song discussions. Although the precise mechanism is not clear, studies have supported evidence that it can induce neurochemical changes and even lead to neural plasticity.

*Occupational therapy/Model of Occupational-Self-Efficacy* (32)

Therapies that improve, maintain and restore an injured or ill individual’s ability to engage in occupations such as work related activities, leisure and self-care.

*Physical therapy/physical rehabilitation* (18)

Any therapy using exercise or body movement that is believed to promote ‘brain health’ and has effects on brain structure and cognitive function.

*Psychological interventions* (33)

Interventions that train people with mental disorders in strategies to manage their symptoms and to identify and challenge patterns of negative thinking. Can range from interventions that are behavioural, cognitive or a combination, and include therapies such as cognitive behavioural therapy, psychotherapy, narrative therapy and so on.

*Qigong* (34)

An ancient Chinese healing art which consists of three basic components: controlled breathing, meditation, and smooth, continuous, purposeful movement. It has been shown to reduce sympathetic activation and enhance cardiovagal tone. It is also believed to promote beneficial changes in the central nervous system.

*Real-life activities in rehabilitation* (35)

Using pre-injury activities and interests as part of rehabilitation.

*Return to work rehabilitation* (6, 36)

Rehabilitation with a specialist (usually an Occupational therapist) that facilitates a patient’s ability to return to their previous work or occupation.

*Smart phone* (37)

Using the smartphone as a tool for memory rehabilitation through audio and visual reminders, Internet-calendars, and also teaching patients how to retrieve information from phones.

*Telerehabilitation/telephone contact with patients* (38, 39)

The utilisation of telecommunication and information systems to deliver rehabilitative care to patients from a distance.

*Trabajadora de salud* (40)

Solution focused brief therapy delivered by lay health workers. This goal-oriented therapy which is an approach to psychotherapy allows the patient to focus on the present and future circumstances and goals, rather than past experiences.

*Working Memory Training* (41)

An experimental training based on the repeated administration of the Paced Auditorily Serial Addition Test where patients would have to add each new number in the sequence of digits to the immediately preceding one and say the answer aloud.

*Yoga* (42)

A physical, mental and spiritual discipline that originated in India and has roots in Hinduism, Buddhism and Jainism. It is believed to influence neurophysiological pathways that can lead to change in emotions and cognition.

*References:*

1. Wong V, Cheuk DKL, Lee S, Chu V. Acupuncture for acute management and rehabilitation of traumatic brain injury. *Cochrane Database of Systematic Reviews.* 2011; [doi.org/10.1002/14651858.CD007700.pub2](https://doi.org/10.1002/14651858.CD007700.pub2)
2. Stapleton M. Effectiveness of Animal Assisted Therapy after brain injury: A bridge to improved outcomes in CRT. *NeuroRehabilitation*. 2016;39: 135-140.
3. Howie P. *Art Therapy with Trauma*. In: Gussak DE, Rosal ML. *The Wiley Handbook of Art Therapy*. West Sussex: John Wiley and Sons; 2016. p.375-386.
4. Kline T. Art therapy for Individuals with Traumatic Brain Injury: A comprehensive neurorehabilitation-informed approach to treatment. *Art Therapy*. 2016;33(2): 67-73.
5. Whitfield G, Davidson AJW. *Cognitive Behavioural Therapy Explained*. Oxford: Radcliffe Publishing Ltd; 2007.
6. Scheenen ME, Visser-Keizer A, van der Naalt, J, Spikman JM. Description of an early cognitive behavioural intervention (UPFRONT-intervention) following mild traumatic brain injury to prevent persistent complaints and facilitate return to work. *Clinical Rehabilitation*. 2017;31(8): 1019-1029.
7. Parola A, Bosco FM, Gabbatore I, Galetto V, Zettin M, Marini A. The impact of the Cognitive Pragmatic Treatment on the pragmatic and informative skills of individuals with traumatic brain injury (TBI). *Journal of Neurolinguistics*. 2019;51: 53-62.
8. Cooper DB, Bowles AO, Kennedy JE, Curtiss G, French LM, Tate DF, et al. Cognitive Rehabilitation for Military Service Members with Mild Traumatic Brain Injury: A Randomized Clinical Trial. *Journal of Head Trauma Rehabilitation*. 2017;32 (3): E1-E15.
9. De Luca R, Portaro S, Le Cause M, De Domenico C, Maggio MG, Cristina Ferrera M, et al. Cognitive rehabilitation using immersive virtual reality at young age: A case report on traumatic brain injury. *Applied Neuropsychology: Child*. 2019. [doi.org/10.1080/21622965.2019.1576525](https://doi.org/10.1080/21622965.2019.1576525)
10. Klonoff PS, O’Brien K, Prigatano GP, Chiapello DA, Cunningham M. Cognitive retraining after traumatic brain injury and its role in facilitating awareness. *Journal of Head Trauma Rehabilitation*. 1989;4: 37-45.
11. Connor BB, Shaw C. Case study series using brain-training games to treat attention and memory following brain injury. *Journal of Pain Management*. 2016;9(3): 217-226.
12. Fernández E, Bringas ML, Salazar S, Rodriguez D, Garcia ME, Torres M. Clinical Impact of RehaCom Software for Cognitive Rehabilitation of Patients with Acquired Brain Injury. *MEDICC Review*. 2012;14(4): 32-35.
13. Robertson, I. Does computerized cognitive rehabilitation work? A review. *Aphasiology.* 1990;4(4): 381-405.
14. Isaacson BM, Swanson TM, Pasquina PF. The use of computer-assisted rehabilitation environment (CAREN) for enhancing wounded warrior rehabilitation. *Journal of Spinal Cord Medicine*. 2013;36(4): 296-299.
15. Chua KS, Kong KH. Rehabilitation outcome following traumatic brain injury-the Singapore experience. *International Journal of Rehabilitation Research*. 1999;22(3): 189-197.
16. Swaine BR, Pless IB, Friedman DS, Montes JL. Effectiveness of a head injury program for children: a preliminary investigation. *American Journal of Physical Medicine and Rehabilitation*. 2000;79(5): 412-420.
17. Fogelman D, Zafonte R. Exercise to Enhance Neurocognitive Function after Traumatic Brain Injury. *The American Academy of Physical Medicine and Rehabilitation*. 2012;4: 908-913.
18. Morris T, Osman JG, Munoz JMT, Miserachs DC, Leone AP. The role of physical exercise in cognitive recovery after traumatic brain injury: A systematic review. *Restorative Neurology and Neuroscience*. 2016;34: 977-988.
19. Lindsay S, Hartman LR, Reed N, Gan C, Thomson N, Solomon B. A Systematic Review of Hospital-to-School Reintegration Interventions for Children and Youth with Acquired Brain Injury. *PLoS ONE*. 2015;10(4): e0124679.
20. Wood, A. Rehabilitation of executive function deficits following brain injury: a randomised controlled trial using Goal Management Training and Implementation Intentions to improve prospective memory [dissertation]. Glasgow: University of Glasgow; 2011.
21. Tiwari D, Daly, C, Alsalaheen B. Home-based circuit training program for an adolescent female with severe traumatic brain injury: A case report. *Physiotherapy Theory and Practice*. 2018;34(2): 137-145.
22. Keegan LC, Murdock M, Suger C, Togher L. Improving natural social interaction: Group rehabilitation after Traumatic Brain Injury. *Neuropsychological Rehabilitation*. 2019. [doi.org/10.1080/09602011.2019.1591464](https://doi.org/10.1080/09602011.2019.1591464)
23. Bédard M, Felteau M, Marshall S, Cullen N, Gibbons C, Dubois S, et al. Mindfulness-Based Cognitive Therapy Reduces Symptoms of Depression in People With a Traumatic Brain Injury: Results from a Randomized Controlled Trial. *Journal of Head Trauma Rehabilitation*. 2014;29(4): e13-e22.
24. Solana J, Cáceres C, Garcia-Molina A, Chausa P, Opisso E, Roig-Rovira T, et al. Intelligent Therapy Assistant (ITA) for cognitive rehabilitation in patients with acquired brain injury. *BMC Medical Informatics and Decision Making*. 2014;14(58).
25. Nelson LA, MacDonald M, Stall C, Pazdan R. Effects of Interactive Metronome Therapy on Cognitive Functioning After Blast-Related Brain Injury: A Randomized Controlled Pilot Trial. *Neuropsychology*. 2013;27(6): 666-679.
26. Bay E, Chan R, Grimm C. Complex intervention development: A positive focused mindfulness group intervention compared to healthy living after TBI*. Journal of Psychosocial Nursing and Mental Health Services*. 2019;57(1): 26-33.
27. Coombs MA. Relax while you rehabilitate: A pilot study integrating a novel, yoga-based mindfulness group intervention into a residential military brain injury rehabilitation program. *Rehabilitation Psychology*. 2018;63(2): 182-193.
28. Hedge S. Music-based cognitive remediation therapy for patients with traumatic brain injury. *Frontiers in Neurology*. 2014;5(34): 1-7.
29. Magee WL, Clark I, Tamplin J, Bradt J. Music interventions for acquired brain injury. *Cochrane Database of Systematic Reviews.* 2017;1. [doi.org/10.1002/14651858.CD006787.pub3](https://doi.org/10.1002/14651858.CD006787.pub3)
30. Nayak S, Wheeler BL, Siflett SC, Agostinelli S. Effect of Music Therapy on Mood and Social Interaction Among Individuals With Acute Traumatic Brain Injury and Stroke. *Rehabilitation Psychology*. 2000;45(3): 274-283.
31. Thaut MH, Gardiner JC, Holmberg D, Horwitz J, Kent L, Andrews G, et al. Neurologic Music Therapy Improves Executive Function and Emotional Adjustment in Traumatic Brain Injury Rehabilitation. *Annals of New York Academy of Science*. 2009;1169: 406-416.
32. Soeker S. The use of Model of Occupational Self Efficacy in improving the cognitive functioning of individuals with brain injury: A pre-and post-intervention study. *Work*. 2017;58: 63-72.
33. Gertler P, Tate RI, Cameraon ID. Non-pharmacological interventions for depression in adults and children with traumatic brain injury. *Cochrane Database of Systematic Reviews*. 2015;12. [doi.org/10.1002/14651858.CD009871.pub2](https://doi.org/10.1002/14651858.CD009871.pub2)
34. Yost TL, Taylor AG. Qigong As A Novel Intervention For Service Members With Mild Traumatic Brain Injury. *US Army Research*. 2013;236: 142-149.
35. Sullivan CT, Gray MA, Williams GP, Green DJ, Hession CA. The Use of Real Life Activities in Rehabilitation: The Experience of Young Men with Traumatic Brain Injuries from Regional, Rural and Remote Areas in Australia. *Journal of Rehabilitation Medicine*. 2014;46: 424-429.
36. Hoosan M. A qualitative investigation of the Individual’s Lived Experience of Returning to Work Following Traumatic Brain Injury [dissertation]. Brighton: University of Brighton; 2010.
37. Evald L. Prospective memory rehabilitation using smartphones in patients with TBI. *Neuropsychological Rehabilitation*. 2015;25(2): 283-297.
38. Betts S, Feichter L, Klenig Z, O’Connell-Debais A, Thai H. Telerehabilitation versus Standard Care for Improving Cognitive Function and Quality of Life for Adults with Traumatic Brain Injury: A Systematic Review. *Internet Journal of Allied Health Sciences and Practice*. 2018;16 (3).
39. Bombadier CH, Bell KR, Tenkin NR, Fann JR, Hoffman J, Dikmen S. The Efficacy of a Scheduled Telephone Intervention for Ameliorating Depressive Symptoms During the First Year After Traumatic Brain Injury. *Journal of Head Trauma Rehabilitation*. 2009;24(4): 230-238.
40. Linton KF, Kim BJ. A pilot study of Trabajadora de salud, a lay health worker intervention for Latinas/os with traumatic brain injuries and their caregivers. *Disability and Health Journal*. 2018;11(1): 161-164
41. Serino A, Ciaramelli E, Santantonio AD, [Malagù S](https://www.ncbi.nlm.nih.gov/pubmed/?term=Malag%C3%B9%20S%5BAuthor%5D&cauthor=true&cauthor_uid=17364515), [Servadei F](https://www.ncbi.nlm.nih.gov/pubmed/?term=Servadei%20F%5BAuthor%5D&cauthor=true&cauthor_uid=17364515), [Làdavas E](https://www.ncbi.nlm.nih.gov/pubmed/?term=L%C3%A0davas%20E%5BAuthor%5D&cauthor=true&cauthor_uid=17364515). A pilot study for rehabilitation of central executive deficits after traumatic brain injury. *Brain Injury*. 2007;21(1): 11-19.
42. Samuel G. The Origins of Yoga and Tantra: Indic religions to the Thirteenth century. Cambridge: Cambridge University Press; 2008.
